# *Faecalibacterium prausnitzii* ameliorates sepsis-induced acute lung injury via gut-lung axis by regulating arachidonic acid-LXA4-Nrf2-HO-1 pathway and restoring gut microecology

**DOI:** 10.3389/fmicb.2026.1820338

**Published:** 2026-05-28

**Authors:** Xiaoli Feng, Pu Xu, Huifang Li, Li Peng, Shan Li, Jie Ma, Junbai Ma, Zongjun Ma, Libing Ma, Ke Li, Tian Ma, Jiayu Yu, Xiaolong Ma, Suzhen Ma, Xiaoxia Zhang, Hao Wang, Xiaojun Yang

**Affiliations:** 1 General Hospital, First Clinical Medical College, Ningxia Medical University, Yinchuan, China; 2 College of Traditional Chinese Medicine, Ningxia Medical University, Yinchuan, China; 3 First People's Hospital of Yinchuan, Yinchuan, China; 4 Ningxia Key Laboratory of Infection and Immunity, School of Basic Medical Sciences, Ningxia Medical University, Yinchuan, China; 5 People's Hospital of Ningxia Hui Autonomous Region, Third Clinical Medical University, Ningxia Medical University, Yinchuan, China; 6 People's Hospital of Litong District, Wuzhong, China

**Keywords:** *F. prausnitzii*, sepsis-induced acute lung injury, gut microbiota, Nrf2-HO-1 pathway, oxidative stress, probiotics

## Abstract

**Introduction:**

Sepsis-induced acute lung injury (S-ALI) is a life-threatening condition in intensive care units. The gut-lung axis has recently emerged as an important regulator of pulmonary inflammation. However, the role of *Faecalibacterium prausnitzii* (*F. prausnitzii*) in S-ALI remains unclear.

**Methods:**

In this study, 16S rRNA high-throughput sequencing revealed significant gut microbiota dysbiosis in S-ALI patients, characterized by a markedly reduced relative abundance of *F. prausnitzii*. Moreover, the depletion of this probiotic was associated with increased clinical inflammatory indices and disease severity. Subsequently, the effects of *F. prausnitzii* strain intervention on S-ALI were investigated in two S-ALI murine models, including cecal ligation puncture (CLP) and lipopolysaccharide (LPS)-induced models.

**Results:**

We found that *F. prausnitzii* intervention significantly attenuated acute lung injury in sepsis by reducing inflammation and oxidative stress. Importantly, *F. prausnitzii* treatment restored gut microbial homeostasis by enhancing intestinal barrier integrity and increasing the production of short-chain fatty acids (SCFAs), particularly butyrate. Mechanistically, non-targeted metabolomics analysis revealed that the protective effects of *F. prausnitzii* may be associated with the modulation of arachidonic acid (AA) metabolism, accompanied by increased levels of the anti-inflammatory mediator lipoxin A4 (LXA4). In addition, the expression of Nrf2 and HO-1 was upregulated in the intervention group, suggesting a potential involvement of the LXA4-Nrf2-HO-1 signaling pathway. Correlation analysis further indicated complex interactions among gut microbiota, SCFAs, metabolomic profiles, and inflammatory and oxidative stress-related factors following *F. prausnitzii* intervention.

**Discussion:**

Collectively, *F. prausnitzii* ameliorates the progression of S-ALI by restoring gut microecology and may be associated with AA metabolism and oxidative stress-related pathways, which may contribute to a potential therapeutic strategy targeting the gut-lung axis and AA metabolic pathways.

## 1 Introduction

Sepsis defined as life-threatening organ dysfunction caused by a dysregulated host response to infection is an important global health problem (Singer et al., 2016). According to statistics released by the Global Burden of Disease Study, there were over 166 million cases of sepsis worldwide in 2021, with sepsis-related deaths accounting for nearly 31.5% of all global deaths (Rudd et al., 2020). Organ injury is a common complication of sepsis, with the lungs being the most frequently affected organ, and approximately 45% of sepsis patients progress to acute respiratory distress syndrome (ARDS) and acute lung injury (ALI) (Kumar, 2020). The pathophysiological process of acute lung injury in sepsis is complex, involving excessive inflammatory response, exacerbated oxidative stress, impaired lung barrier function, and vascular leakage (Xu et al., 2024b). Despite significant advances in understanding these mechanisms, no specific clinical treatments are currently available, and management remains limited to symptomatic support. Therefore, identifying novel therapeutic targets for S-ALI is critical.

The gut, as the largest microbial ecosystem in the human body maintains a delicate balance of its microbial community that is closely linked to immune function and inflammatory responses (Campbell et al., 2023). Dysbiosis of the gut microbiota has been demonstrated to be associated with the onset or progression of various diseases, including S-ALI, obesity, and Kawasaki disease (KD) (Jena et al., 2025; Li et al., 2025; Saad and Santos, 2025). Increasing evidence indicates that sepsis is frequently associated with profound gut microbiota dysbiosis, characterized by reduced microbial diversity, including decreased richness and evenness, which reflects severe disruption of intestinal microbial homeostasis (Liu et al., 2023; Yi et al., 2026). In addition to the loss of diversity, compositional alterations are also observed, typically characterized by a depletion of beneficial commensal bacteria such as *F. prausnitzii, Bifidobacterium*, and *Lactobacillus*, alongside expansion of opportunistic pathogens including members of *Enterococcus, Escherichia*, and other *Proteobacteria* (Chen et al., 2024; Ren et al., 2024; De Gaay Fortman et al., 2026). These microbial alterations are often accompanied by impaired intestinal barrier function, commonly referred to as “leaky gut”. Disruption of the intestinal barrier allows harmful substances, such as bacterial toxins, including LPS, to translocate into the bloodstream. This not only triggers localized inflammation in the gut but may also induce inflammatory responses in distant organs like the lungs. Consequently, gut microbiota dysbiosis and barrier dysfunction significantly increase the risk of S-ALI (Chen et al., 2025; Zhao and Wu, 2026).

Among next-generation probiotics, *F. prausnitzii* has garnered significant attention. This commensal bacterium is abundant in the gut microbiota of healthy adults and is recognized for its butyrate production and associated anti-inflammatory and immunomodulatory properties (Quévrain et al., 2016; Munukka et al., 2017; Lenoir et al., 2020; Shi et al., 2025). However, the alleviating effect of *F. prausnitzii* on S-ALI remains largely unexplored.

Arachidonic acid (AA) is an important omega-6 long-chain polyunsaturated fatty acid (PUFA). The gut microbiota regulate PUFA metabolism, and in turn, PUFAs influence gut microbiota composition and diversity, as well as intestinal inflammation (Bascuñán et al., 2025). AA is hydrolyzed by phospholipase A2 (PLA2) on the inner surface of the cell membrane to its free form, and is subsequently metabolized via cyclooxygenases (COXs), lipoxygenases (LOXs), and cytochrome P450 (CYP) enzymes to generate multiple bioactive mediators, including prostaglandins, leukotrienes (LTs), and lipoxins (LXs) (Wang et al., 2021). AA and its metabolites have been extensively studied in the fields of cardiovascular disease and cancer biology, where they play critical roles in inflammatory processes and disease pathogenesis. Evidence indicates that AA and its downstream anti-inflammatory metabolite LXA4 exert protective effects against acute lung injury (ALI). For instance, in mice fed a high-fat diet, AA alleviates LPS-induced inflammatory responses in macrophages and reduces mortality in septic mice by binding to MD2 (Zhang et al., 2020). In a mouse model of LPS-induced ALI, after 6 weeks of AA supplementation, analysis of bronchoalveolar lavage fluid and lung tissue revealed that AA significantly attenuated alveolar septal thickness and bronchoalveolar area (Molchanova et al., 2022). LXA4 alleviates LPS-induced and transfusion-associated ALI in mice by modulating neutrophil-platelet aggregation (Ortiz-Muñoz et al., 2014). Furthermore, LXA4 increases the activity of antioxidant enzymes in various organs and plays a protective role in the restoration of redox homeostasis (Jiang, 2023; Rizk et al., 2023; Xu et al., 2024a).

Despite growing evidence linking gut microbiota dysbiosis to the pathogenesis of S-ALI, the underlying mechanisms remain poorly understood. In particular, the potential role of *F. prausnitzii*, a key commensal bacterium with anti-inflammatory properties, in modulating S-ALI has yet to be fully elucidated. In this study, we aimed to investigate whether *F. prausnitzii* exerts protective effects against S-ALI and to explore the underlying mechanisms, with a particular focus on gut microbiota regulation, intestinal barrier function, and AA-related metabolic pathways, potentially involving oxidative stress-related processes.

## 2 Materials and methods

### 2.1 Clinical research

Based on the SEPSIS 3.0 diagnostic criteria and S-ALI criteria, fecal samples were collected from 15 patients with S-ALI admitted to the Department of Critical Care Medicine at Ningxia Medical University General Hospital. Ten healthy donors were enrolled as healthy control (HC). These samples underwent 16S rRNA high-throughput sequencing. The inclusion criteria for S-ALI patients were as follows: age ≥18 years; diagnosis of sepsis according to the Sepsis-3.0 criteria (Singer et al., 2016) and S-ALI criteria (ARDS Definition Task Force et al., 2012). The exclusion criteria were as follows: patients with perianal infections; patients who have undergone colostomy; patients with chronic gastrointestinal diseases; diagnosis of hematologic or immunologic disorders; patients receiving immunosuppressants or chemotherapy. Additionally, the requirements for the healthy control group were as follows: age matched with the S-ALI group patients; healthy physical condition; no history of chronic or metabolic diseases (such as hypertension, coronary heart disease, diabetes, hepatitis, or hyperthyroidism); no history of gastrointestinal diseases or gastrointestinal surgeries; no use of antibiotics, probiotics, enteral nutrition, or other medications within 3 months prior to enrollment. The clinical study was approved by the Ethics Committee of Ningxia Medical University General Hospital (Approval No: 2019-386).

Clinical data of S-ALI patients were collected, including age, sex, primary diagnosis, BMI and underlying diseases. Acute Physiology and Chronic Health Evaluation II (APACHE II) and Sequential Organ Failure Assessment (SOFA) scores were documented on the day of ICU admission (Supplementary Table 1). Blood samples, fecal samples, or rectal swabs were collected from patients in the intensive care unit (ICU) prior to antibiotic administration at admission. Clinical parameters were recorded, including serum albumin (ALB), white blood cell count (WBC), neutrophil percentage (NEUT%), lymphocyte percentage (LYM%), hemoglobin (Hb), platelet count (PLT), procalcitonin (PCT), urea (Urea), creatinine (Cr), aspartate transaminase (AST), alanine transaminase (ALT), S/F ratio (S/F), lactate (Lac), prothrombin time (PT), and prothrombin activity (PTA). These indicators were used for subsequent correlation analysis with gut microbiota.

### 2.2 Culture of *F. prausnitzii*

*F. prausnitzii* (ATCC 27768) was firstly activated on ATCC Medium 260 (Tryptic Soy Medium with 5% Defibrinated Sheep Blood) agar plates. The activated *F. prausnitzii* strain was then inoculated into an liquid medium with a 1% (v/v) inoculum and incubated at 37 °C for 72h in a rocking bed. Bacterial cells were collected via centrifugation (4,000 × g, 5min, 4 °C). The cell pellet was washed twice with sterile normal saline (0.9% NaCl) and subsequently resuspended in sterile normal saline for oral administration to mice.

### 2.3 Experimental animals

All animal experiments were approved by the Ethics Committee of Ningxia Medical University (IACUC-2024041). This study utilized male C57BL/6 mice (aged 6–8 weeks, weight 20 ± 2 g) that were purchased from Vital River Laboratory Animal Technology Co., Ltd. in Beijing, China. These mice were group-housed under standard specific pathogen-free (SPF) conditions in a temperature-regulated environment (22 ± 1 °C, 40–70 % humidity) with a 12-h light/dark cycle at the Laboratory Animal Center of Ningxia Medical University in Yinchuan, China, and had *ad libitum* access to food and water.

### 2.4 Animal treatment and experimental design

In this study, two sepsis murine models were induced by both CLP operation representing a classic sepsis model that mimics polymicrobial infection and exacerbates the systemic inflammatory response, and LPS administration displaying an another sepsis model that mimics gram-negative bacterial infection and can stably induce hyper-inflammatory responses.

After 1 week of adaptation, mice were randomly divided into four groups (*n* = 10 per group): the sham group (Sham), the cecal ligation and puncture-induced S-ALI group (CLP), the *F. prausnitzii* sham group (FP) and the *F. prausnitzii* treatment group (CLP_FP). To investigate the effect of *F. prausnitzii* on S-ALI, mice in the FP and CLP_FP were pretreated with *F. prausnitzii* (2 × 10^9^CFU/ml) by oral gavage for 4 weeks. Mice in the sham and CLP groups were given the same dose of sterile normal saline.

Following this, mice in the *F. prausnitzii* and CLP groups underwent cecal ligation and puncture to induce S-ALI. Briefly, mice were anesthetized by intraperitoneal injection of pentobarbital sodium (50 mg/kg), followed by disinfection of the surgical area and a 1–1.5 cm incision along the abdominal midline to expose the cecum. We performed a tight ligation at the midpoint of the cecum. Then, two sides of the bowel wall were punctured with an 21G needle, squeezed out 1–2 drops of cecal contents. After the cecum was returned to the abdominal cavity, the abdominal wall was closed in layers using 4-0 surgical sutures. Sham-operated mice underwent laparotomy and bowel manipulation without ligation and puncture. After surgery, all mice were revived via subcutaneous injection of warm sterile normal saline and maintained warm using an electric heating pad. At 24 h after surgery, mice were deeply anesthetized with pentobarbital sodium, and tissue collection was performed under deep anesthesia; animals did not regain consciousness during the procedure.

For LPS-induced lung injury, mice were intraperitoneally injected (i.p.) with LPS at a dose of 10 mg/kg. Mice were randomly divided into four groups (*n* = 10 per group): the control group (CON), the LPS-induced acute lung injury group (LPS), the *F. prausnitzii* negative control group (FP), and the *F. prausnitzii* treatment group (LPS_FP). Gastrointestinal pre-treatment was performed identically to that in the CLP model. After 24 h, mice were deeply anesthetized, and tissue samples were collected under anesthesia following the same procedures as those described for the CLP model.

### 2.5 Measurement of the lung wet-to-dry (W/D) weight ratio

Immediately following dissection, the wet weights of right lower lobe tissues were recorded, followed by dehydration in an 65 °C oven until constant weight was achieved. The wet-to-dry weight ratios were calculated to assess edema.

### 2.6 Collection of bronchoalveolar lavage fluid (BALF)

After anesthesia with pentobarbital sodium, the mice were euthanized, and BALF was collected by gently washing the lungs three times with 1 ml of PBS. After centrifugation of the BALF at 1,000 × g for 5 min at 4 °C, the supernatant was used to measure protein concentration via the BCA assay kit (KeyGEN BioTECH, KGB2101-250). The cell pellet was resuspended in 100 μl PBS, and cell counts in BALF were determined using an automated cell counter (JIMBIO iCytal S2).

### 2.7 Histological analysis

Lung, small intestine and colon tissue fragments were fixed in 4% paraformaldehyde for 24 h, followed by graded dehydration and embedded in paraffin. Tissue sections (5 μm) were stained with hematoxylin-eosin and dehydrated with an ethanol gradient, xylene transparent twice and sealed with neutral resin. The morphology and structure of the tissues were examined under a light microscope, and the lung injury score (LIS) was quantified in a blinded manner (Matute-Bello et al., 2011).

### 2.8 Quantification of *F. prausnitzii* colonization

Total genomic DNA was extracted from fecal samples using a Stool DNA Kit (Omega Bio-tek, Norcross, GA, USA; Cat. No. D4015) according to the manufacturer's instructions. The abundance of *F. prausnitzii* was quantified by qPCR using species-specific primers and normalized to total bacterial 16S rRNA gene levels, qPCR was performed using TB Green Premix Ex Taq II (Takara Bio Inc., Shiga, Japan), and relative abundance was calculated using the 2^∧^–ΔΔCt method.

### 2.9 Quantitative real-time PCR (RT-qPCR)

Total RNA was extracted from lung tissue using an RNA Extraction Kit (Omega, USA) according to the manufacturer's protocol. Complementary DNA (cDNA) was synthesized by reverse transcription using the PrimeScript™ FAST RT reagent Kit with gDNA Eraser (Takara Bio Inc., Japan). Quantitative real-time PCR (RT-qPCR) was performed using TB Green Premix Ex Taq II (Takara Bio Inc., Shiga, Japan). The relative expression levels of each mRNA were normalized to the internal reference gene GAPDH and calculated using the 2^∧^–ΔΔCt method. Primer sequences (Sangon Biotech, Shanghai, China) were shown in Table 1.

**Table 1:** Primer sequences used for gene expression analysis and bacterial quantification.

Gene	Primers	Sequences (5^′^to 3^′^)
Gene expression analysis	
TNF-α	Forward	GCGACGTGGAACTGGCAGAAG
	Reverse	GCCACAAGCAGGAATGAGAAGAGG
IL-6	Forward	GAGAGGAGACTTCACAGAGGATACC
	Reverse	TCATTTCCACGATTTCCCAGAGAAC
IL-10	Forward	TCCCTGGGTGAGAAGCTGAAGAC
	Reverse	CACCTGCTCCACTGCCTTGC
IL-1β	Forward	TCGCAGCAGCACATCAACAAG
	Reverse	AGGTCCACGGGAAAGACACAG
Nrf2	Forward	GACGGGACTATTGAAGGCTGTGAC
	Reverse	TGGGACTCGTGTTCAGTGAAATGC
HO-1	Forward	ACCGCCTTCCTGCTCAACATTG
	Reverse	CTCTGACGAAGTGACGCCATCTG
LXA4	Forward	TTGCCTTGGACCGCTGTATTTG
	Reverse	AAATCCAGGACCCAACAACCAC
Bacterial quantification	
16S rRNA (total bacteria)	Forward	ACTCCTACGGGAGGCAGCAG
	Reverse	ATTACCGCGGCTGCTGG
*F. prausnitzii*	Forward	GGAGGAAGAAGGTCTTCGG
	Reverse	AATTCCGCCTACCTCTGCACT

### 2.10 Measurement of superoxide dismutase (SOD) and malondialdehyde (MDA) in lung tissue

Lung tissues were mechanically homogenized and centrifuged at 12,000 × g for 10 min at 4 °C. The resulting supernatant was collected, and protein concentrations were quantified using a BCA protein assay kit. Levels of SOD and MDA in lung tissue were then measured using commercial assay kits in accordance with the manufacturer's protocols (Nanjing Jiancheng Bioengineering Institute, Nanjing, China).

### 2.11 Measurement of plasma LPS Level

Plasma LPS level was measured using a Limulus amebocyte lysate (LAL) assay kit according to the manufacturer's instructions (Xiamen Bioendo Technology Co., Ltd., Xiamen, China). Mouse plasma samples were first centrifuged at 3,000 × g for 10 min, and the supernatants were transferred to pyrogen-free EP tubes for subsequent use. A standard curve was generated using serial dilutions of bacterial endotoxin standards (0.1–1.0 EU/ml). Standards or samples were incubated with LAL reagent at 37 °C, followed by chromogenic reaction and termination according to the kit protocol. Absorbance was measured at 545 nm using a microplate reader, and LPS concentrations were calculated based on the standard curve (*R*^2^ > 0.99).

### 2.12 Enzyme-linked immunosorbent assay (ELISA)

The levels of plasma inflammatory cytokines including interleukin-6 (IL-6), interleukin-1β (IL-1β), and tumor necrosis factor-α (TNF-α) and interleukin-10 (IL-10) were determined using ELISA kits (Wuhan SanYing Biotechnology Co., Ltd., Wuhan, China), following the manufacturer's instructions.

### 2.13 Tissue immunofluorescence staining

Paraffin sections were deparaffinized and rehydrated, followed by antigen retrieval in sodium citrate buffer using microwave heating at medium–high power for 18 min. Sections were blocked with goat serum at 37 °C for 1 h and then incubated overnight at 4 °C in the dark with primary antibodies against occludin (sc-133256, Santa Cruz Biotechnology), claudin-4 (sc-376643, Santa Cruz Biotechnology), or ZO-1 (sc-33725, Santa Cruz Biotechnology). After washing with phosphate-buffered saline (PBS), sections were incubated with goat anti-mouse FITC-conjugated secondary antibody (GB22301, Servicebio) for occludin and claudin-4, or goat anti-rat Cy3-conjugated secondary antibody (GB21302, Servicebio) for ZO-1 at 37 °C for 1 h. Nuclei were counterstained with DAPI (G1410, Servicebio), and sections were mounted with antifade mounting medium and examined under a fluorescence microscope.

For double-label three-color immunofluorescence staining, sections were deparaffinized, rehydrated, and subjected to antigen retrieval as described above. Subsequent staining procedures were performed according to the manufacturer's instructions of the Tyramide signal amplification (TSA) Fluorescence Double Staining Kit (G1226, Servicebio, China). Stained sections were subsequently examined using a confocal laser scanning microscope.

### 2.14 Non-targeted metabolomics analysis

Fecal samples were thawed at 4 °C and vortexed for 1 min to ensure homogeneity. An appropriate amount of each sample was accurately transferred into a 2 ml centrifuge tube, followed by the addition of 400 μl methanol. The mixture was vortexed for 1 min and centrifuged at 12,000 rpm for 10 min at 4 °C. The supernatant was carefully collected, transferred into a new 2 ml centrifuge tube, and concentrated to dryness. The dried extract was reconstituted in 150 μl of 2-chloro-L-phenylalanine (4 ppm) solution prepared in 80% methanol–water. After centrifugation, the supernatant was filtered through a 0.22 μm membrane and transferred into LC–MS vials for untargeted metabolomics analysis. To ensure analytical stability and data quality, pooled quality control (QC) samples were prepared by mixing equal aliquots of all samples and were injected periodically throughout the analytical run, while 2-chloro-L-phenylalanine was used as an internal standard to monitor extraction efficiency and instrument performance.

Liquid chromatography–mass spectrometry (LC–MS) analysis was performed using a Vanquish UHPLC system coupled with a Q Exactive mass spectrometer (Thermo Fisher Scientific, USA) equipped with an electrospray ionization (ESI) source. Chromatographic separation was achieved on an ACQUITY UPLC^®^ HSS T3 column (2.1 × 100 mm, 1.8 μm; Waters, Milford, MA, USA) maintained at 40 °C, with a flow rate of 0.3 ml/min and an injection volume of 2 μl. For ESI positive-ion mode, the mobile phases consisted of 0.1% formic acid in water (A2) and 0.1% formic acid in acetonitrile (B2); for ESI negative-ion mode, the mobile phases were 5 mM ammonium formate in water (A3) and acetonitrile (B3). The gradient elution program was as follows: 0–1 min, 8% B; 1–8 min, 8–98% B; 8–10 min, 98% B; 10–10.1 min, 98–8% B; 10.1–12 min, 8% B. Mass spectrometric data were acquired in both positive and negative ion modes using full MS/ddMS2 acquisition. The spray voltage was set at 3.50 kV for ESI (+) and −2.50 kV for ESI (–), with sheath gas at 40 arb and auxiliary gas at 10 arb. The capillary temperature was maintained at 325 °C. Full MS scans were acquired at a resolution of 70,000 over an m/z range of 100–1,000, followed by data-dependent MS/MS scans of the top 10 ions at a resolution of 17,500 using higher-energy collisional dissociation (HCD) with a normalized collision energy of 30 eV and dynamic exclusion enabled.

### 2.15 Gut microbiota analysis

Fecal microbial 16S rRNA gene sequencing and analysis were carried out following established methods. The fecal sample was suspended in 790 μl of sterile lysis buffer [4M guanidine thiocyanate; 10% N-lauroyl sarcosine; 5% N-lauroyl sarcosine-0.1 M phosphate buffer (pH 8.0)] in 2 ml screw-cap tube containing 1g glass beads (0.1mm BioSpec Products, Inc., USA). This mixture was vortexed vigorously and then incubate at 70 °C for 1h. After incubation by bead beating for 10min at maximum speed. DNA was extracted by following the manufacturer's instructions for bacterial DNA extraction using The E.Z.N.A.^®^ Genomic DNA was extracted using a Stool DNA Kit (Omega Bio-tek, Inc., GA, USA) according to the manufacturer's instructions, with the lysis step omitted. The extracted DNA was stored at −20 °C for further analysis. The extracted DNA from each sample was used as the template to amplify the V3-V4 region of 16S rRNA genes. The primers F1 and R2 (5′-CCTACGGGNGGCWGCAG-3′ and 5′-GACTACHVGGGTATCTAATCC-3′) correspond to positions 341 to 805 in the 16S rRNA gene were used to amplify the V3-V4 region of each fecal sample by PCR. PCR reactions were run in a EasyCycler 96 PCR system (Analytik Jena AG, Jena, Germany) using the following program: 3 min of denaturation at 95 °C followed by 21 cycles of 0.5 min at 94 °C (denaturation), 0.5 min for annealing at 58 °C, and 0.5 min at 72 °C (elongation), with a final extension at 72 °C for 5 min. The products from different samples were indexed and mixed at equal ratios for sequencing by Shanghai Mobio Biomedical Technology Co. Ltd. using the NextSeq 2000 platform (Illumina Inc., USA) according to the manufacturer's instructions.

High-quality reads were processed using an ASV-based workflow implemented in DADA2 for error correction and inference of exact sequence variants. Taxonomic classification was performed by aligning ASV representative sequences against the SILVA SSU138 database. To account for differences in sequencing depth, α-diversity analyses were conducted using rarefied ASV tables. For mouse samples, rarefaction was performed at 32,000 reads per sample, whereas for clinical samples, a rarefaction depth of 30,000 reads per sample was used. Rarefaction curves indicated that sequencing depth was sufficient for both datasets, as most curves approached a plateau, capturing the majority of microbial diversity. Alpha diversity indices including Chao1, ACE and Shannon were calculated using mothur v1.42.1, and data visualization and statistical analyses were performed in R using the phyloseq and vegan packages.

Between-sample community dissimilarities were calculated based on Bray–Curtis as well as weighted and unweighted UniFrac distances, and ordination was visualized through principal coordinate analysis (PCoA) and non-metric multidimensional scaling (NMDS). NMDS was performed based on the Bray–Curtis dissimilarity matrix using the vegan package in R, and the stress value was used to evaluate the goodness of fit. Statistical separation of microbial community structures across groups was evaluated using permutational multivariate analysis of variance (PERMANOVA) with 10,000 permutations. In addition, taxonomic composition bar plots from the phylum to genus levels were generated in R, and differences in relative abundances of bacterial taxa were assessed using the non-parametric Mann–Whitney *U*-test for comparisons between two groups, with false discovery rate (FDR) adjustment, and the Kruskal–Wallis rank-sum test for comparisons among three or more groups. The false discovery rate (FDR) *q*-value was calculated for the *p*-values. Differentially enriched taxa were identified using LEfSe (Linear Discriminant Analysis Effect Size) analysis, conducted using the Galaxy web-based platform (https://github.com/SegataLab/lefse). The LDA score threshold was set to 3.0 to identify significantly discriminative features between groups. Statistical significance was determined based on a *p*-value threshold of 0.05. The key ASVs with significant differences between groups were selected at the ASV level using a random forest classifier model, and abundance heatmaps were generated.

### 2.16 Determination of SCFAs in mice stool

Standard solutions of acetic acid, propionic acid, butyric acid and isovaleric acid were prepared in water at a concentration of 100 mg/ml, while hexanoic acid was prepared in methyl tert-butyl ether (MTBE) at 100 mg/ml. Serial dilutions were used to obtain working standard solutions. The internal standard, 4-methylvaleric acid, was prepared in MTBE at 375 μg/ml. Calibration standards were prepared by mixing 200 μl of working standards for the six SCFAs, 20 μl of hexanoic acid working standard, 100 μl of 15% phosphoric acid, 20 μl of internal standard solution, and 260 μl of MTBE, yielding ten calibration points ranging from 0.02 to 500 μg/ml. Stock solutions were stored at −20 °C, and working solutions were freshly prepared before analysis.

Fecal samples were weighed and transferred into 1.5 ml centrifuge tubes, followed by the addition of 500 μl distilled water and 100 mg glass beads. Samples were homogenized for 1 min and centrifuged at 12,000 rpm for 10 min at 4 °C. Subsequently, 200 μl of the supernatant was mixed with 100 μl of 15% phosphoric acid, 20 μl of internal standard solution (4-methylvaleric acid, 375 μg/ml), and 280 μl of MTBE, homogenized for 1 min, and centrifuged again at 12,000 rpm for 10 min at 4 °C. The organic supernatant was subjected to GC–MS analysis using a 7890A gas chromatography system coupled with a 5975C mass spectrometer (Agilent Technologies, USA) equipped with an HP-INNOWAX capillary column (30 m × 0.25 mm i.d. × 0.25 μm). Samples were injected in split mode (10:1, 1 μl), with an injector temperature of 250 °C and helium as the carrier gas at a flow rate of 1.0 ml/min. The oven temperature was programmed from 90 °C to 120 °C at 10 °C/min, to 150 °C at 5 °C/min, and finally to 250 °C at 25 °C/min with a 2 min hold. Mass spectrometry was performed in electron impact (EI) mode at 70 eV using selected ion monitoring (SIM).

### 2.17 Statistical analysis

Data are presented as mean ± standard error of the mean (SEM), and data analysis and figure generation were performed using Excel and GraphPad Prism 10 (GraphPad Software Inc., CA, USA). According to the normal distribution, the differences between two groups were analyzed using a two-tailed Student's *t*-test. Statistical differences among experimental groups were evaluated using one-way analysis of variance (ANOVA) followed by Tukey's multiple comparison test. Correlation analysis was performed using Spearman's rank method. For non-targeted metabolomics analysis, MetaboAnalyst (www.metaboanalyst.ca) was used to perform KEGG pathway enrichment analysis of the list of differential metabolites (Xia and Wishart, 2011). For multiple comparisons, the pairwise *t*-test was applied when homogeneity of variance was satisfied, whereas the Games–Howell test was used when variances were unequal. A *p* value < 0.05 was considered statistically significant.

## 3 Results

### 3.1 Lack of intestinal *F. prausnitzii* participates in the progression of S-ALI patients

**Figure 1:**
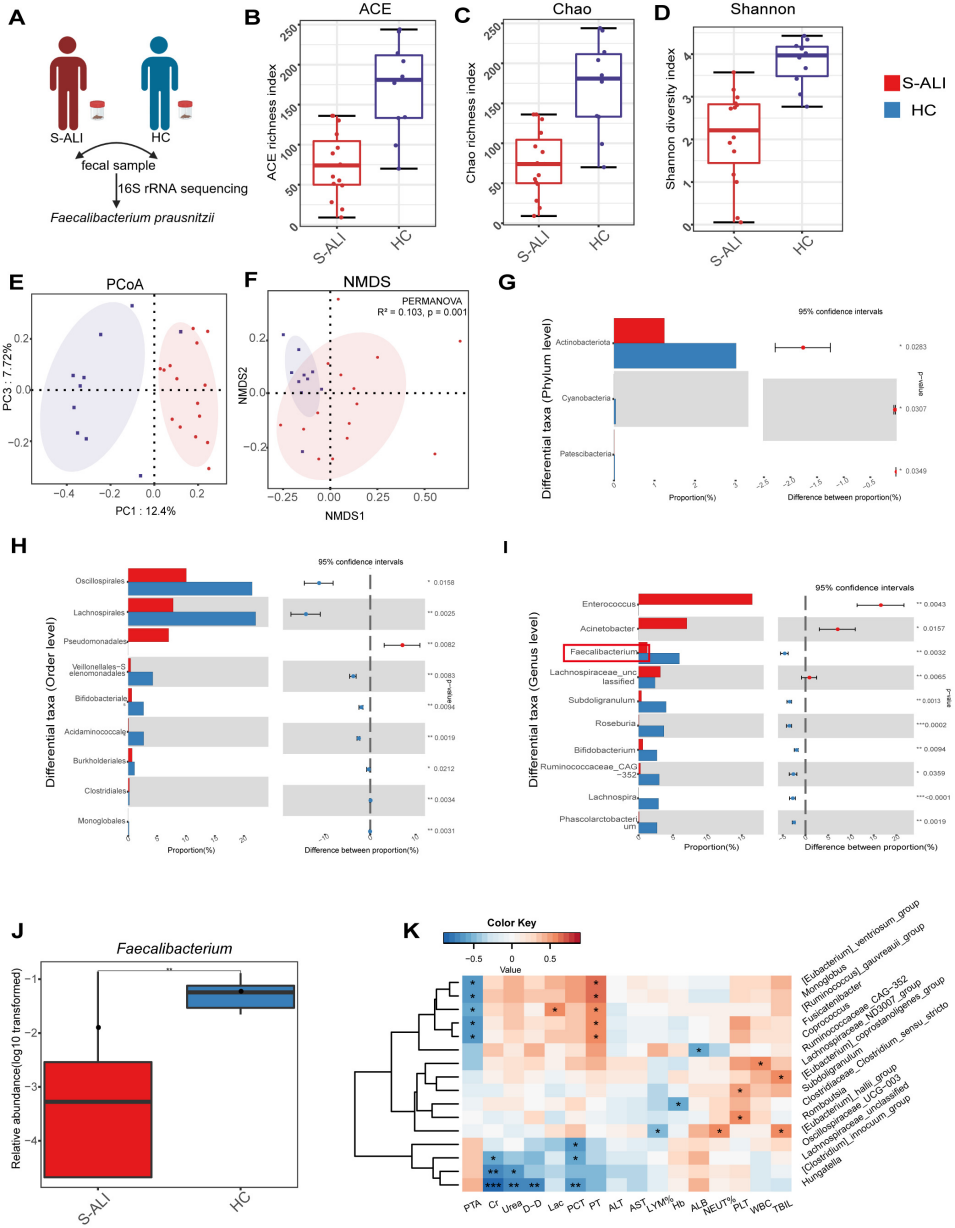
Dysbiosis of gut microbiota in clinical patients with S-ALI. **(A)** Schematic illustration of fecal sample collection from patients with S-ALI (*n* = 15 per group) and HC (*n* = 10 per group) for 16S rRNA gene sequencing analysis. Alpha diversity of gut microbiota assessed by the ACE **(B)**, Chao **(C)**, and Shannon **(D)** indices. **(E–F)** Beta diversity analysis (PCoA and NMDS) of gut microbiota. Differential abundance analysis of the gut microbiota between S-ALI and HC across multiple taxonomic levels. Relative abundance and proportion differences with 95% confidence intervals are shown at the phylum **(G)**, order **(H)**, and genus **(I)** levels. Relative abundance of *Faecalibacterium* in fecal samples **(J)**. Heatmap of correlations between selected gut microbial genera and clinical parameters **(K)**, illustrating significant associations between microbial alterations and disease-related clinical indices. *p < 0.05, **p < 0.01, ***p < 0.001.

We performed 16S rRNA high-throughput sequencing on fecal samples from patients with S-ALI and healthy controls to investigate potential associations between the gut microbiota and S-ALI development (Figure 1A). Alpha diversity reflects the abundance and diversity of the gut microbiota. Results showed that compared to the HC group, the S-ALI group exhibited significantly reduced α-diversity metrics (Ace index, Chao index, and Shannon index; Figures 1B–D). Rarefaction curves for all clinical samples reached a plateau, indicating that the sequencing depth was adequate to capture the majority of microbial diversity (Supplementary Figure S1). These findings suggest that the observed differences in α-diversity between groups were not influenced by sequencing depth, indicating markedly weakened gut microbial diversity in patients with sepsis-associated acute lung injury. β-diversity was assessed using principal coordinate analysis (PCoA) and non-metric multidimensional scaling (NMDS) based on Bray–Curtis dissimilarity (Figures 1E, F). Both analyses showed a clear separation between the S-ALI and HC groups. Permutational multivariate analysis of variance (PERMANOVA) confirmed that microbial community composition differed significantly between groups (*R*^2^ = 0.103, *p* = 0.001).

At the phylum level, the gut microbiota of both groups was dominated by *Actinobacteriota*. Compared to healthy controls, the relative abundance of *Actinobacteriota* was significantly reduced in the S-ALI group (difference: approximately −1.8% in relative abundance, *p* = 0.0283). In addition, *Cyanobacteria* (*p* = 0.0307) and *Patescibacteria* (*p* = 0.0349) also showed statistically significant differences between groups, although their relative abundances were extremely low (Figure 1G). At the class level, S-ALI patients exhibited a significantly increased relative abundance of *Clostridia* (*p* = 0.0044) and *Actinobacteria* (*p* = 0.0315), while *Negativicutes* (*p* < 0.0001) was markedly reduced compared with HC (Supplementary Figure S2). At the order level, S-ALI patients exhibited a significant reduction in *Oscillospirales* (*p* = 0.0158) and *Lachnospirales* (*p* = 0.0025), along with an enrichment of *Pseudomonadales* (*p* = 0.0082) compared with HC, indicating a substantial alteration in gut microbial composition (Figure 1H). At the genus level, S-ALI patients showed a pronounced enrichment of potentially pathogenic genera, including *Enterococcus* (*p* = 0.0043) and *Acinetobacter* (*p* = 0.0157). In contrast, SCFAs-producing and beneficial genera including *F. prausnitzii* (*p* = 0.0032), *Lachnospiraceae_unclassified* (*p* = 0.0065), *Subdoligranulum* (*p* = 0.0013), *Roseburia* (*p* = 0.0002), *Bifidobacterium* (*p* = 0.0094), *Ruminococcaceae_CAG-352* (*p* = 0.0359), *Lachnospira* (*p* < 0.0001), and *Phascolarctobacterium* (*p* = 0.0019), were significantly depleted in S-ALI patients compared with HC (Figure 1I). Given its known anti-inflammatory properties, *F. prausnitzii* was further analyzed. The relative abundance of *Faecalibacterium* was dramatically reduced in S-ALI patients compared with HCs (Figure 1J), highlighting a potential role of this genus in the pathogenesis of S-ALI. The relative abundance of key taxa across phylum, class, order, and genus levels, along with the corresponding SEM, is summarized in Supplementary Table 2.

Finally, spearman correlation analysis revealed strong associations between gut microbiota alterations and clinical parameters. Beneficial SCFAs-producing genera, including *Subdoligranulum*, and *Roseburia*, were negatively correlated with inflammatory markers (CRP, PCT, WBC, and NEUT%) and tissue injury indicators (LDH and lactate). In contrast, opportunistic pathogens *Enterococcus* and *Acinetobacter* were positively correlated with systemic inflammation and organ dysfunction indices (Figure 1K). These findings suggest that gut microbiota dysbiosis is closely linked to immune dysregulation and disease severity in S-ALI. In summary, these results suggest that *F. prausnitzii* may play a crucial role in this study, particularly in relation to the maintenance of gut microbial homeostasis and immune regulation. Based on its significant alterations, subsequent studies will further focus on the biological functions of *F. prausnitzii* and its potential therapeutic value.

### 3.2 *F. prausnitzii* protects against S-ALI in two murine CLP models

**Figure 2:**
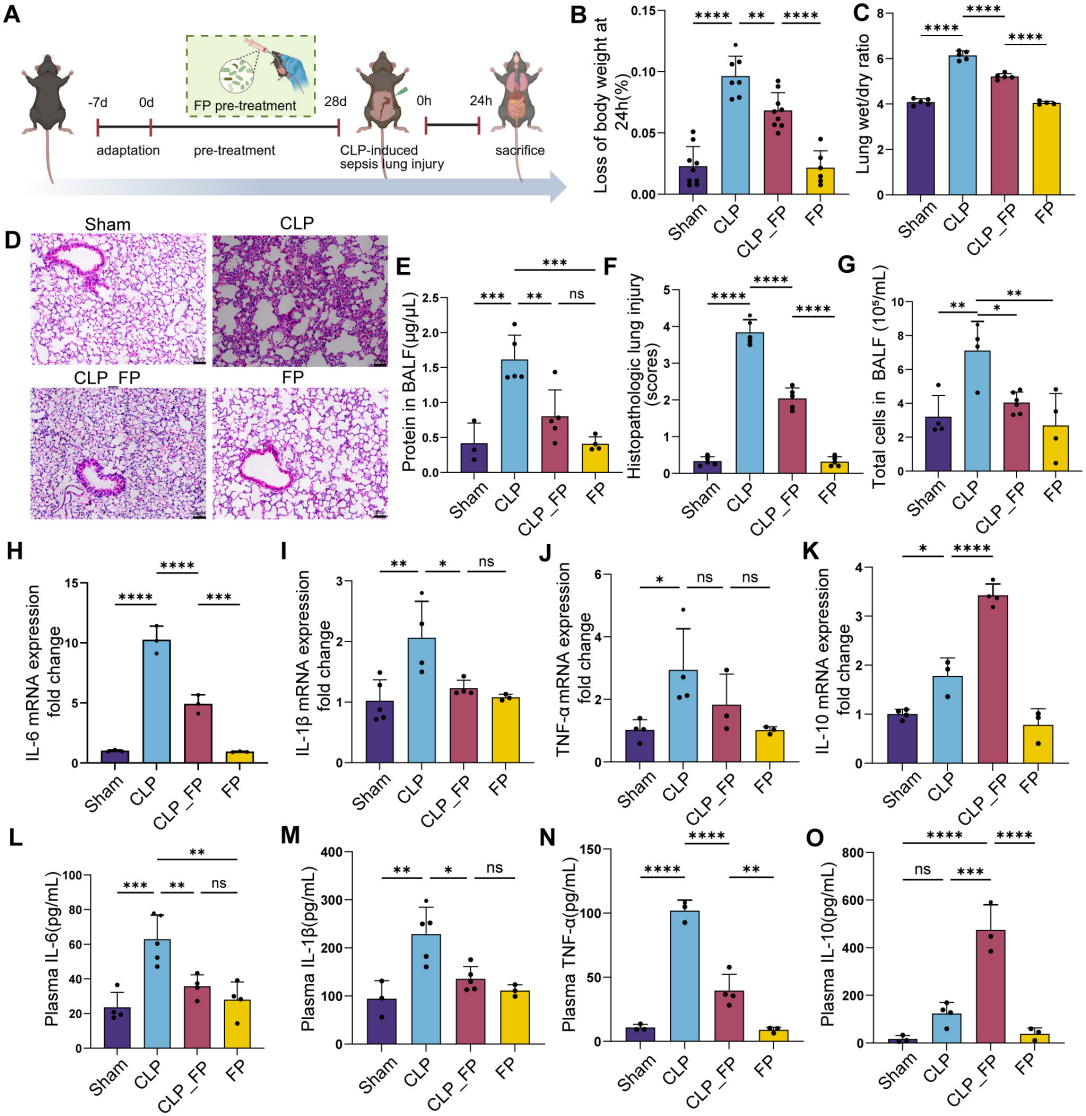
*F. prausnitzii* pre-treatment ameliorates CLP induced lung injury. **(A)** Showing a schematic diagram of live *F. prausnitzii* pre-treatment CLP mice. **(B)** Loss body weight at 24 h after surgery (*n* = 6–9 per group), **(C)** lung wet/dry weight ratio (n = 4–5 per group). **(D, F)** Representative H&E staining and damage scores of lung tissue (scale bar = 50 μm). **(E)** Protein concentration in BALF (*n* = 3–5 per group). **(G)** Total cell counts in BALF (*n* = 4–5 per group). **(H–K)** mRNA expression levels of inflammatory cytokines IL-6, IL-1β, TNF-α and IL-10 in lung tissues (*n* = 3–5 per group). **(L–O)** Protein levels in plasma of inflammatory cytokines IL-6, IL-1β, TNF-α and IL-10 (*n* = 3–5 per group). *p < 0.05, **p < 0.01, ***p < 0.001, ****p < 0.0001, ns, no significance.

To clarify the direct effect of *F. prausnitzii* on S-ALI, mice were administered live *F. prausnitzii* by oral gavage for 1 month before the CLP procedure (Figure 2A). After confirming successful colonization of *F. prausnitzii* in the gut through qPCR analysis (Supplementary Figure S3), the mice underwent CLP surgery to induce S-ALI. Firstly, compared to the control group, the CLP group exhibited significantly reduced body weight loss at 24 h post-modeling, while *F. prausnitzii* pretreatment resulted in a higher weight loss than before (Figure 2B). Similarly, the lung wet-to-dry weight ratio was markedly increased following CLP challenge, indicating pulmonary edema, which was significantly reduced by *F. prausnitzii* pre-treatment (Figure 2C). We subsequently assessed protein concentration in BALF, a key indicator of alveolar-capillary barrier permeability. Compared to controls, the CLP group exhibited significantly higher protein levels, suggesting increased pulmonary permeability (Figure 2E). Notably, live *F. prausnitzii* treatment markedly attenuated protein leakage, further supporting its protective role in S-ALI.

Histopathological examination of lung tissue via H&E staining revealed significant pathological alterations in the CLP group compared to the control group, including disruption of lung architecture, thickening of alveolar septa, inflammatory cell infiltration in alveolar spaces and interstitial areas, alveolar hemorrhage, and elevated lung injury scores (Figures 2D, F). Notably, live *F. prausnitzii* significantly attenuated these pathological alterations. These findings indicate that live *F. prausnitzii* not only improves vascular permeability in mice with acute septic lung injury but also mitigates lung tissue damage.

### 3.3 *F. prausnitzii* alleviates inflammation in CLP mice with S-ALI

In the S-ALI mouse model, immune cells such as neutrophils accumulate and become activated within lung tissue, thereby exacerbating the inflammatory response. At the end of the animal experiment, we measured the total cell counts levels in BALF. Results showed a significant increase in total cell counts in BALF from the sepsis group. However, pretreatment with live *F. prausnitzii* significantly reduced the total cell counts in BALF from the sepsis group (Figure 2G).

Subsequently, we delved into the detailed mechanisms underlying the anti-sepsis effects of live *F. prausnitzii*. Inflammation (manifesting as a cytokine storm) is one of the primary factors causing cell death during the progression of sepsis. Therefore, we focused on whether live *F. prausnitzii* plays a crucial role in mitigating the severity of excessive inflammation during acute lung injury in sepsis. To investigate this phenomenon, we employed RT-qPCR to detect the expression levels of inflammatory cytokine mRNA in lung tissue. Results revealed significantly elevated levels of pro-inflammatory factors—including IL-6, IL-1β, and TNF-α–in septic mice, alongside markedly reduced levels of the anti-inflammatory factor IL-10 (Figures 2H–K). However, pretreatment with live *F. prausnitzii* significantly reduced proinflammatory factor levels and increased anti-inflammatory factor levels in the lung tissue of mice with acute lung injury induced by sepsis.

Additionally, systemic inflammatory responses were observed in the septic mice. We detected inflammatory cytokines in serum and found that live *F. prausnitzii* effectively reduced pro-inflammatory cytokine levels while increasing anti-inflammatory cytokine levels in septic mice (Figures 2L–O). These results indicate that live *F. prausnitzii* simultaneously alleviates both pulmonary and systemic inflammation in mice with acute lung injury induced by sepsis.

### 3.4 Live *F. prausnitzii* improves intestinal mucosal barrier function

Endotoxin LPS triggers a “cytokine storm” in the pulmonary inflammation of sepsis, which is a key factor exacerbating lung injury. We measured LPS level in the systemic circulation of mice and found that serum LPS level was significantly elevated in acute lung injury sepsis mice compared to normal mice. Pretreatment with live *F. prausnitzii* significantly reduced serum LPS levels in acute lung injury sepsis mice.

**Figure 3:**
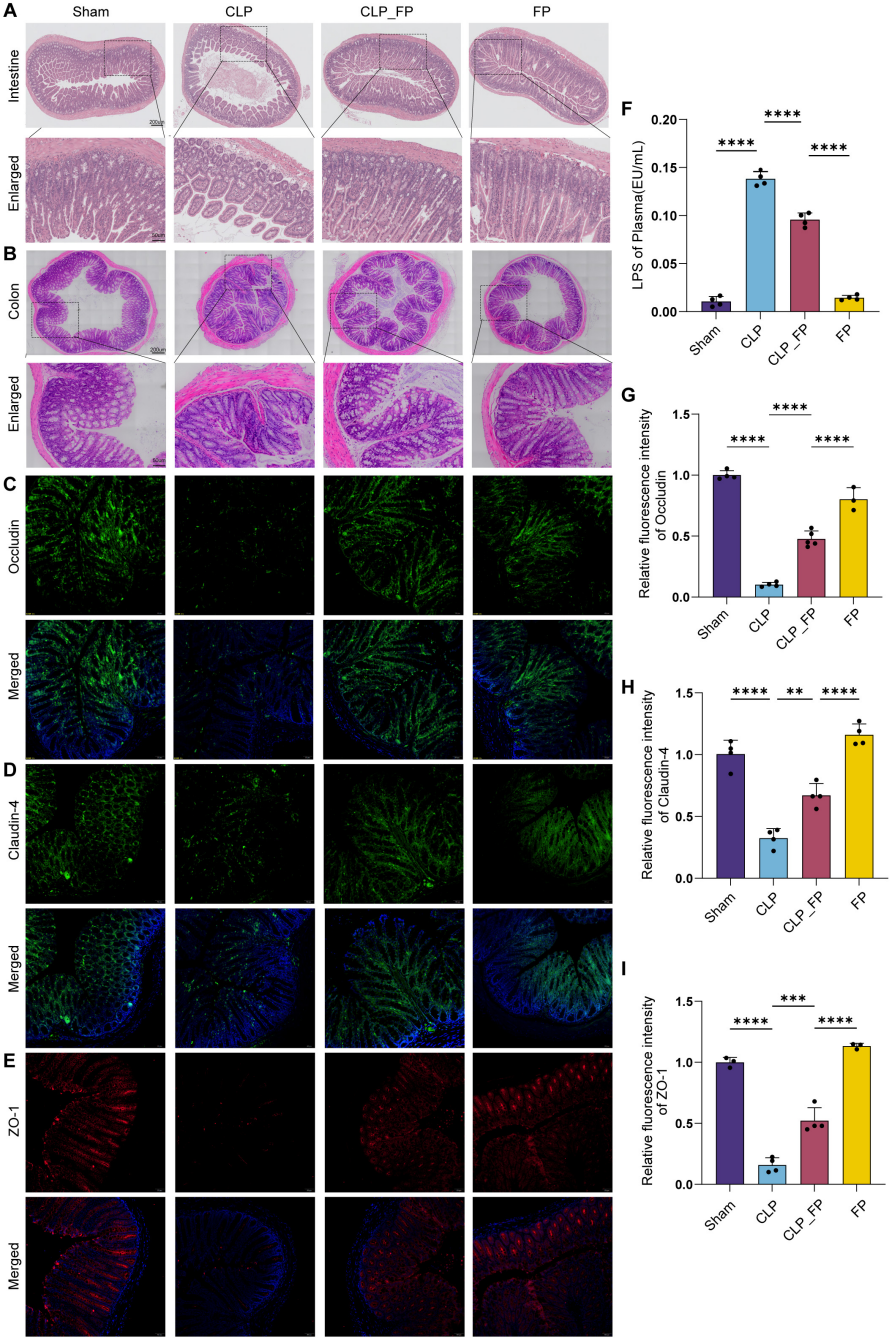
Live *F. prausnitzii* pre-treatment improves intestinal mucosal barrier function. **(A–B)** Representative H&E staining of the small intestine and colon. The scale bar in the upper images represents 200 μm, while the scale bar in the lower images represents 50 μm. These images show the morphological changes observed in the intestinal tissue under different magnifications. **(C–E)** Results are presented as the area ratio (positive staining/total area) for occludin, claudin-4, and ZO-1 (scale bar = 50 μm). **(F)** Plasma LPS level. **(G–I)** Relative fluorescence intensity of occludin, claudin-4 and ZO-1 (*n* = 3–5 per group). Data were presented as mean ± SEM, **p < 0.01, ***p < 0.001, ****p < 0.0001.

A key reason for elevated LPS levels in the circulation during sepsis is compromised intestinal barrier integrity, which increases intestinal permeability and allows enteric LPS to enter the bloodstream. To investigate this phenomenon, we firstly assessed the histology of mouse small and large intestinal tissues. H&E staining revealed mild edema, villous separation, and mucosal damage in the small intestine of mice. These pathological alterations were confirmed in the model group but were absent in the control group. In contrast, compared to the model group, the *F. prausnitzii* intervention group showed improved intestinal damage in the CLP model (Figure 3A). H&E staining revealed normal colonic architecture with intact mucosa in control mice. In contrast, the CLP group exhibited partial crypt damage and inflammatory cell infiltration within colonic tissue (Figure 3B). Following live *F. prausnitzii* pretreatment, intestinal structure in septic mice showed marked improvement compared to the model group, with significantly reduced inflammatory cell infiltration.

To further assess intestinal barrier integrity, we measured the levels of typical tight junction proteins expressed by intestinal epithelial cells (IECs) including ZO-1, Occludin, and Claudin-4. Immunofluorescence results revealed reduced expression of these tight junction proteins in the mucosal epithelial cells of septic mice, accompanied by disruption of the mucosal barrier structure (Figures 3C–E). Following live *F. prausnitzii* pretreatment, the expression levels of ZO-1, Occludin, and Claudin-4 increased, and barrier structure significantly improved (Figures 3G–I). Taken together, these findings indicate that live *F. prausnitzii* effectively improves intestinal mucosal barrier function in a mouse model of sepsis with acute lung injury. This live *F. prausnitzii* mitigates sepsis-induced damage of the intestinal mucosal barrier by improving its permeability and thereby lowering blood levels of LPS, and consequently alleviates LPS-induced pulmonary inflammation.

### 3.5 *F. prausnitzii* protects against S-ALI in a LPS murine model

**Figure 4:**
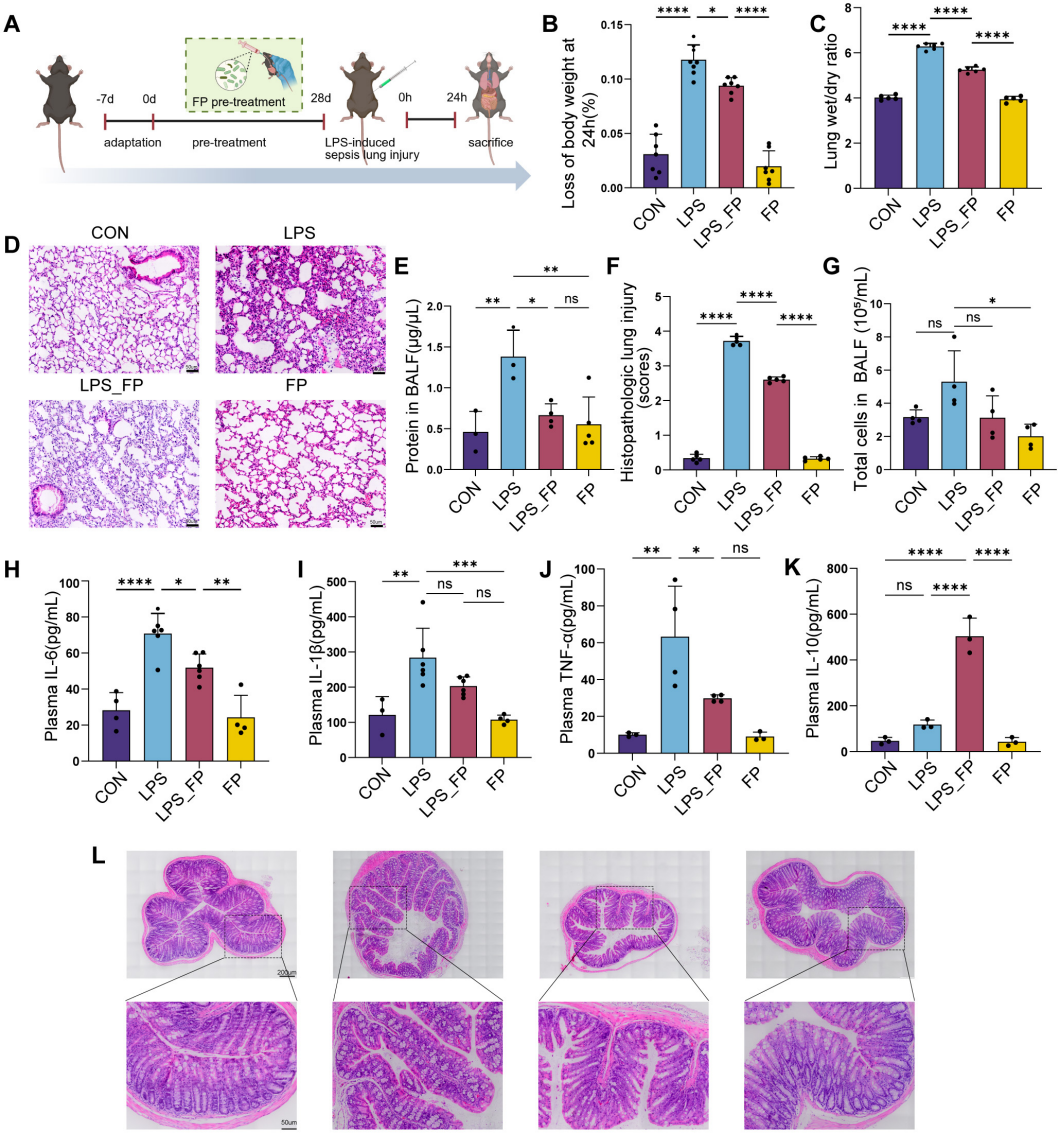
*F. prausnitzii* pre-treatment ameliorates LPS induced lung injury. **(A)** A schematic diagram of Live *F. prausnitzii* pre-treatment LPS mice. **(B)** Loss body weight at 24 h after intraperitoneal Injection LPS (*n* = 6–8 per group). **(C)** The lung wet/dry weight ratios (*n* = 5–6 per group). **(D, F)** Representative H&E staining and damage scores of lung tissue (scale bar = 50 μm). **(E)** Protein concentration in BALF (*n* = 3–5 per group). **(G)** Total cell counts in BALF (*n* = 4 per group). **(H–K)** Inflammatory cytokines IL-6, IL-1β, TNF-α and IL-10 levels in plasma (*n* = 3–6 per group). **(L)** Representative H&E staining of the colon. The scale bar in the upper images represents 200 μm, while the scale bar in the lower images represents 50 μm. These images show the morphological changes observed in the intestinal tissue under different magnifications. Data were presented as mean ± SEM, *p < 0.05, **p < 0.01, ***p < 0.001, ****p < 0.0001, ns, no significance.

The LPS-induced sepsis murine model is a simple model with high reproducibility and reliability that can induce excessive inflammatory responses. To further validate the protective effect of live *F. prausnitzii* on S-ALI, a LPS-induced sepsis-associated acute lung injury model was established (Figure 4A). Parallel experiments showed that an administration of live *F. prausnitzii* significantly attenuated body weight loss at 24 h after LPS challenge (Figure 4B), reduced pulmonary edema (Figure 4C), and decreased lung permeability (Figure 4E). Consistently, the total cell counts in bronchoalveolar lavage fluid were markedly reduced in mice treated with live *F. prausnitzii* (Figure 4G). Histopathological analysis further revealed that lung injury was markedly alleviated in mice treated with live *F. prausnitzii* ( Figures 4D, F). In addition, live *F. prausnitzii* suppressed the expression of proinflammatory factors while enhancing anti-inflammatory cytokines (Figures 4H–K). Moreover, colonic tissue injury was also significantly ameliorated (Figure 4L). Collectively, these findings indicate that live *F. prausnitzii* confers significant protection against LPS-induced acute lung injury in mice.

### 3.6 *F. prausnitzii* modulates gut microbiota of S-ALI mice

**Figure 5:**
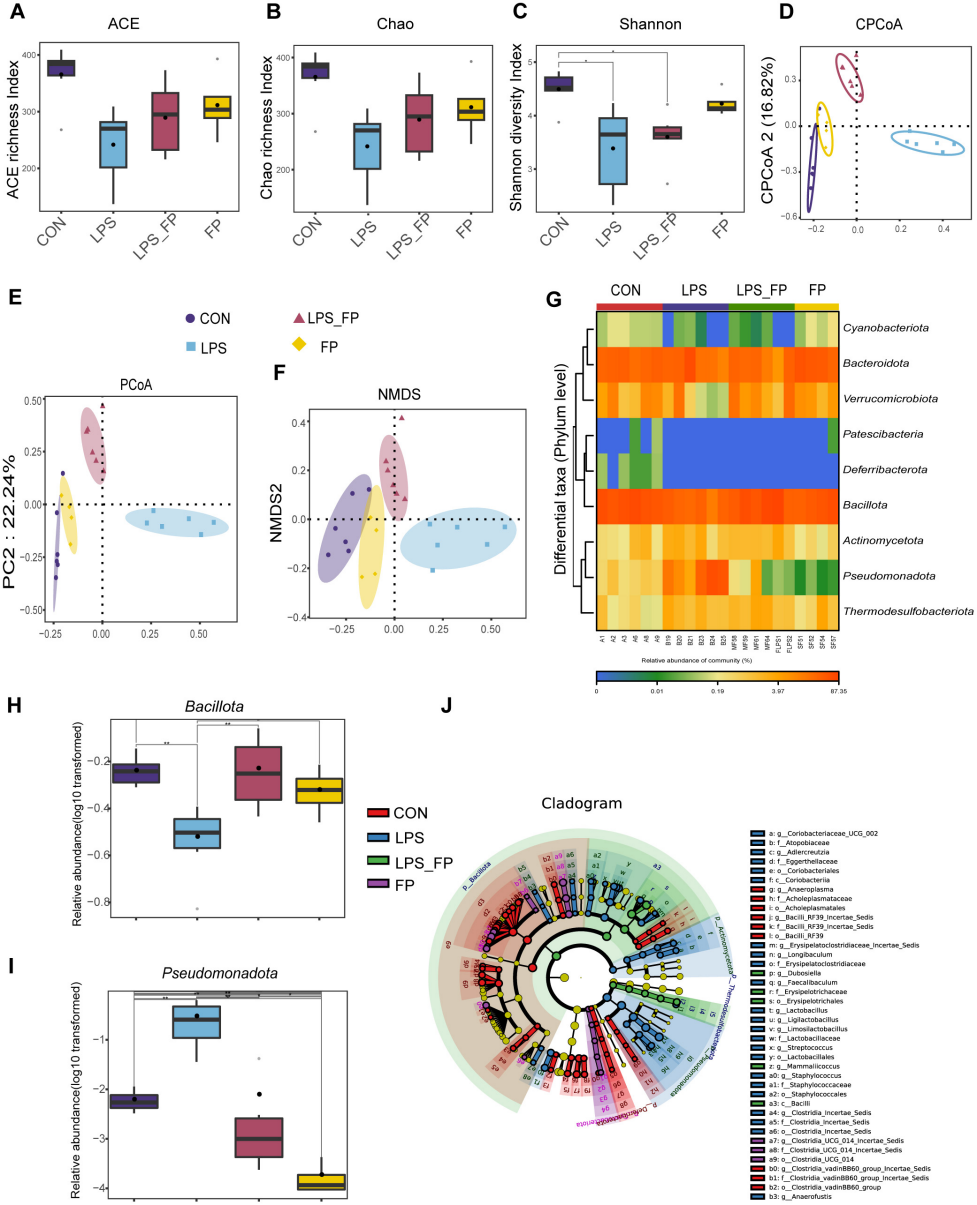
*F. prausnitzii* pre-treatment alters the gut microbiota. Alpha diversity of gut microbiota assessed by the ACE **(A)**, Chao **(B)**, and Shannon **(C)** indices. Beta diversity of the gut microbiota analyzed by constrained principal coordinates analysis (CPCoA) **(D)**, principal coordinates analysis (PCoA) **(E)**, and non-metric multidimensional scaling (NMDS) **(F)**. **(G)** Heatmap illustrating the relative abundance of gut microbial communities at the phylum level across different treatment groups. Relative abundance of representative phylum, including Bacillota **(H)** and Pseudomonadota **(I)**. **(J)** LEfSe cladogram identifying differentially enriched bacterial taxa among groups across multiple taxonomic levels. p* < 0.05, **p < 0.01.

Studies indicate that gut microbiota dysbiosis may be a key factor in the onset and progression of ALI associated with sepsis (Zhao and Wu, 2026). When gut microbiota imbalance occurs, it may trigger abnormal immune responses, leading to intestinal inflammation. Furthermore, overgrowth of certain bacteria may result in the production of harmful metabolites, thereby damaging the intestinal mucosa (Qin et al., 2024). We analyzed intestinal contents via 16S rRNA high-throughput sequencing to investigate gut microbiota composition following live *F. prausnitzii* intervention. Rarefaction curves for all mice samples reached a plateau, indicating that the sequencing depth was adequate to capture the majority of microbial diversity (Supplementary Figure S4). Alpha diversity analysis (Ace index, Chao index, and Shannon index) showed reduced microbial richness and diversity in LPS groups, with partial restoration following *F. prausnitzii* intervention (Figures 5A–C). Beta diversity was employed to compare microbial community composition differences across samples. Beta-diversity analyses based on constrained principal coordinates analysis (CPCoA), non-metric multidimensional scaling (NMDS), and principal coordinate analysis (PCoA) revealed distinct clustering of samples within each treatment group, along with clear separation between different groups, indicating significant differences in gut microbiota composition (Figures 5D–F). Notably, samples from the control group clustered closer to those from the LPS_FP group than to those from the LPS group across all three ordination methods, suggesting that *F. prausnitzii* treatment partially restores the microbial community structure disrupted by sepsis.

**Figure 6:**
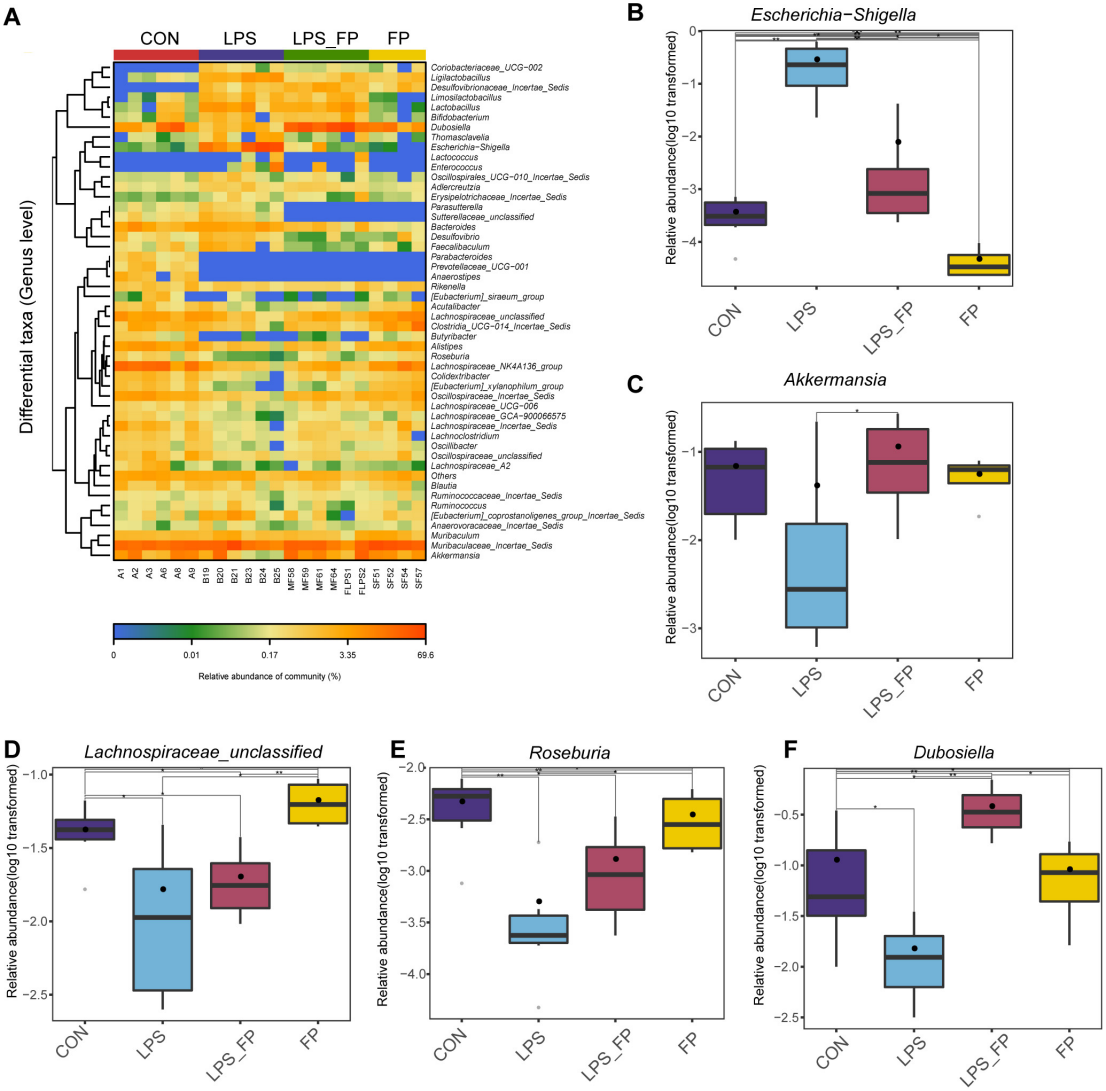
Genus-level alterations of gut microbiota following *F. prausnitzii* pre-treatment. **(A)** Heatmap showing the relative abundance of dominant bacterial genera across different treatment groups. Relative abundance of representative genera, including *Escherichia–Shigella* **(B)**, *Akkermansia* **(C)**, *Lachnospiraceae_unclassified* **(D)**, *Roseburia* **(E)**, and *Dubosiella* **(F)**. *p < 0.05, **p < 0.01.

To further investigate the relative abundance of the gut microbiota following different treatments, gut microbial composition was analyzed at both the phylum and genus levels. The results showed that the bacterial composition of *F. prausnitzii* treated mice differed markedly from that of the other groups, and the relative abundance of characteristic microbial taxa was systematically examined across different taxonomic levels. At the phylum level, LPS challenge markedly altered the overall composition of the gut microbiota, as evidenced by a decreased relative abundance of *Bacillota* and a concomitant expansion of *Pseudomonadota* compared with the control group (Figures 5G–I). Notably, administration of live *F. prausnitzii* partially restored the abundance of *Bacillota* while significantly suppressing the overgrowth of *Pseudomonadota*, indicating a reversal of LPS-induced microbial dysbiosis. Genus-level heatmap analysis revealed that *F. prausnitzii* treated mice maintained a microbial composition more similar to that of the control group. Specifically, *F. prausnitzii* administration increased the abundance of beneficial genera, including *Akkermansia, Lachnospiraceae_unclassified, Roseburia*, and *Dubosiella*, while significantly decreasing *Escherichia–Shigella*, a genus associated with intestinal dysbiosis and inflammatory responses (Figures 6A–F). The LEfSe analysis revealed key discriminative taxa with notable shifts in microbial composition. In the LPS group, *Pseudomonadota* was significantly increased, indicating dysbiosis, while *F. prausnitzii* treatment helped reverse this by reducing *Pseudomonadota* levels. Additionally, *F. prausnitzii* restored the abundance of *Bacillota*, promoting microbial balance. The treatment also enriched beneficial genera, including *Akkermansia, Lachnospiraceae unclassified, Roseburia*, and *Dubosiella*, while significantly decreasing *Escherichia-Shigella*, a genus linked to intestinal dysbiosis (Figure 5J). Collectively, these results suggest that live *F. prausnitzii* partially restores the gut microbial community structure in S-ALI mice toward a homeostatic state, prompting further investigation into whether *F. prausnitzii* associated microbial metabolites, particularly short-chain fatty acids (SCFAs), are involved.

### 3.7 *F. prausnitzii* enhances SCFAs production in S-ALI mice

**Figure 7:**
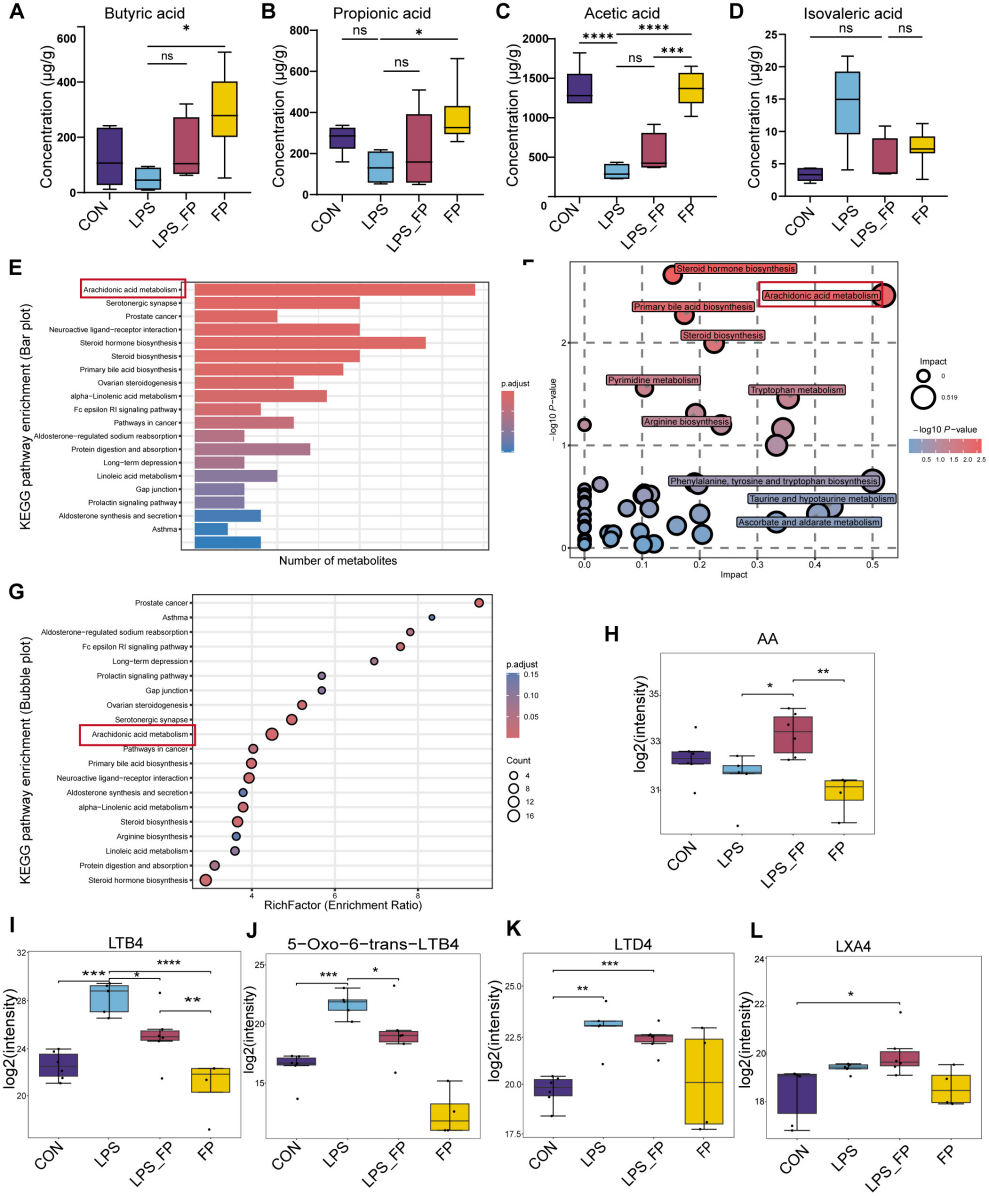
*F. prausnitzii* modulates short-chain fatty acids (SCFAs) and host lipid-related metabolic pathways in S-ALI mice. Relative abundances of major SCFAs, including butyric acid **(A)**, propionic acid **(B)**, acetic acid **(C)**, and isovaleric acid **(D)**, in fecal samples from the CON, LPS, LPS_FP, and FP groups. KEGG enrichment of differentially expressed: bar plot of metabolic pathways **(E)**, scatter plot of metabolic pathways **(F)**, bubble plot of metabolic pathway **(G)**. **(H)** Relative intensity of AA across different treatment groups. Relative intensities of representative AA-derived lipid mediators, including leukotriene B4 (LTB4) **(I)**, 5-oxo-6-trans-LTB4 **(J)**, leukotriene D4 (LTD4) **(K)**, and LXA4 **(L)**. *p < 0.05, **p < 0.01, ***p < 0.001, ****p < 0.0001, ns, no significance.

Given that *F. prausnitzii* is one of the major butyrate-producing commensal bacteria in the gut, we next examined fecal short-chain fatty acid profiles in mice. Compared with the control group, LPS challenge significantly altered the SCFAs profile. Butyric acid and acetic acid levels were markedly reduced in the LPS group, while propionic acid also showed a decreasing trend. *F. prausnitzii* intervention significantly increased butyric acid and acetic acid levels compared with the LPS group, and partially restored their concentrations toward those observed in the control group (Figures 7A, B). Propionic acid was also elevated following *F. prausnitzii* treatment compared with the LPS group (Figure 7C). In contrast, isovaleric acid, a branched-chain SCFAs derived from protein fermentation, was significantly elevated in the LPS group compared with the control group. *F. prausnitzii* intervention showed a decreasing trend in isovaleric acid levels; however, the difference did not reach statistical significance (Figure 7D). These results indicate that *F. prausnitzii* intervention primarily modulated carbohydrate-derived SCFAs, with limited effects on protein fermentation-related SCFAs.

### 3.8 Alterations in the gut metabolites induced by live *F. prausnitzii* intervention in LPS-induced mice

SCFAs, particularly butyrate, are known to exert anti-inflammatory effects and regulate host lipid metabolism (Nireeksha et al., 2025). Given the pronounced changes in fecal SCFAs following *F. prausnitzii* intervention, we next investigated whether *F. prausnitzii* treatment also modulated metabolic pathways. To address this, we employed LC-MS metabolomics technology to identify differential metabolites in fecal samples. To further characterize the classification and functional properties of these metabolites, we performed KEGG pathway annotation and highlighted key metabolic pathways. Analysis using bar charts, bubble charts, and network diagrams revealed that the primary differential metabolites across groups were enriched in pathways including α-Linolenic acid metabolism, primary bile acid biosynthesis, and steroid hormone biosynthesis. Among these pathways, AA metabolism showed both high enrichment significance and pathway impact, highlighting its central role in LPS-induced metabolic alterations (Figures 7E–G).

At the metabolite level, LPS challenge markedly increased fecal levels of pro-inflammatory arachidonic acid-derived lipid mediators, including leukotriene B4 (LTB4), 5-oxo-6-trans-LTB4, and leukotriene D4 (LTD4) (Figures 7I–K). *F. prausnitzii* intervention significantly reshaped arachidonic acid metabolism by increasing fecal AA levels while simultaneously reducing the accumulation of these downstream pro-inflammatory leukotrienes (Figure 7H). Notably, the anti-inflammatory lipid mediator LXA4 was significantly elevated following *F. prausnitzii* treatment compared with the LPS group (Figure 7L). These findings indicate that *F. prausnitzii* intervention reprogrammed AA metabolism toward an anti-inflammatory profile, characterized by reduced production of pro-inflammatory leukotrienes and enhanced generation of inflammation-resolving lipid mediators.

### 3.9 *F. prausnitzii* intervention modulates the gut-lung axis via systemic AA-associated antioxidant responses

**Figure 8:**
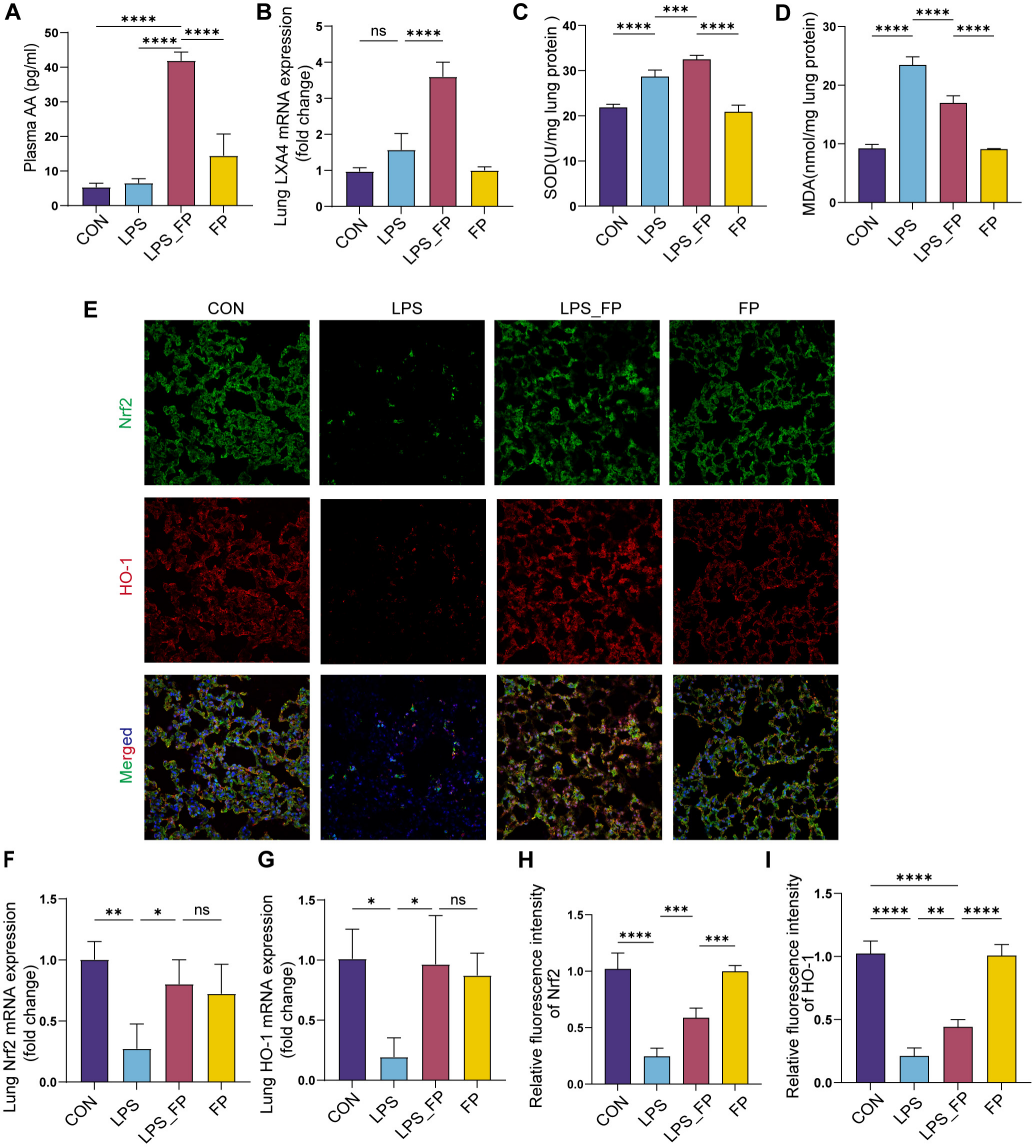
*F. prausnitzii* modulates Nrf2/HO-1 related antioxidant responses in S-ALI mice. **(A)** Levels of AA in plasma measured by ELISA. **(B)** Relative mRNA expression of LXA4. Activities of antioxidant and oxidative stress markers, including superoxide dismutase (SOD) **(C)** and malondialdehyde (MDA) **(D)** in lung tissue. Representative immunofluorescence images showing Nrf2 (**green**) and HO-1 (**red**) expression in lung tissue. Nucleus were counterstained with DAPI (**blue**), (scale bar = 50 μm) **(E)**. Relative mRNA expression levels of Nrf2 **(F)** and HO-1 **(G)**. Quantification of immunofluorescence intensity of Nrf2 **(H)** and HO-1 **(I)**. Data were presented as mean ± SEM (*n* = 3–5 per group). *p < 0.05, **p < 0.01, ***p < 0.001, ****p < 0.0001, ns, no significance.

Given the emerging role of the gut-lung axis in regulating pulmonary inflammation, we next examined whether intestinal metabolic alterations were accompanied by systemic changes. As shown in Figure 8A, *F. prausnitzii* intervention significantly increased plasma AA levels compared with the LPS group, indicating altered systemic availability of gut-associated lipid metabolites. In parallel, *F. prausnitzii* treatment markedly elevated the levels and mRNA expression of the anti-inflammatory lipid mediator LXA4 (Figure 8B). Consistent with these changes, LPS challenge significantly reduced SOD activity and increased MDA levels in lung tissues, whereas *F. prausnitzii* intervention effectively restored SOD activity and attenuated lipid peroxidation (Figures 8C, D).

Furthermore, immunofluorescence staining revealed that *F. prausnitzii* intervention robustly enhanced the expression of Nrf2 and HO-1 in lung tissues compared with the LPS group (Figure 8E). Quantitative analyses confirmed significant upregulation of Nrf2 and HO-1 at both mRNA and protein levels following *F. prausnitzii* treatment (Figures 8F–I). Collectively, these results suggest that *F. prausnitzii* intervention modulates systemic AA-associated metabolic signals along the gut-lung axis, which may be associated with the activation of pulmonary Nrf2-HO-1 antioxidant defenses and the alleviation of LPS-induced oxidative stress.

### 3.10 Correlation analysis among gut microbiota, SCFAs, AA and inflammation, and oxidative stress

**Figure 9:**
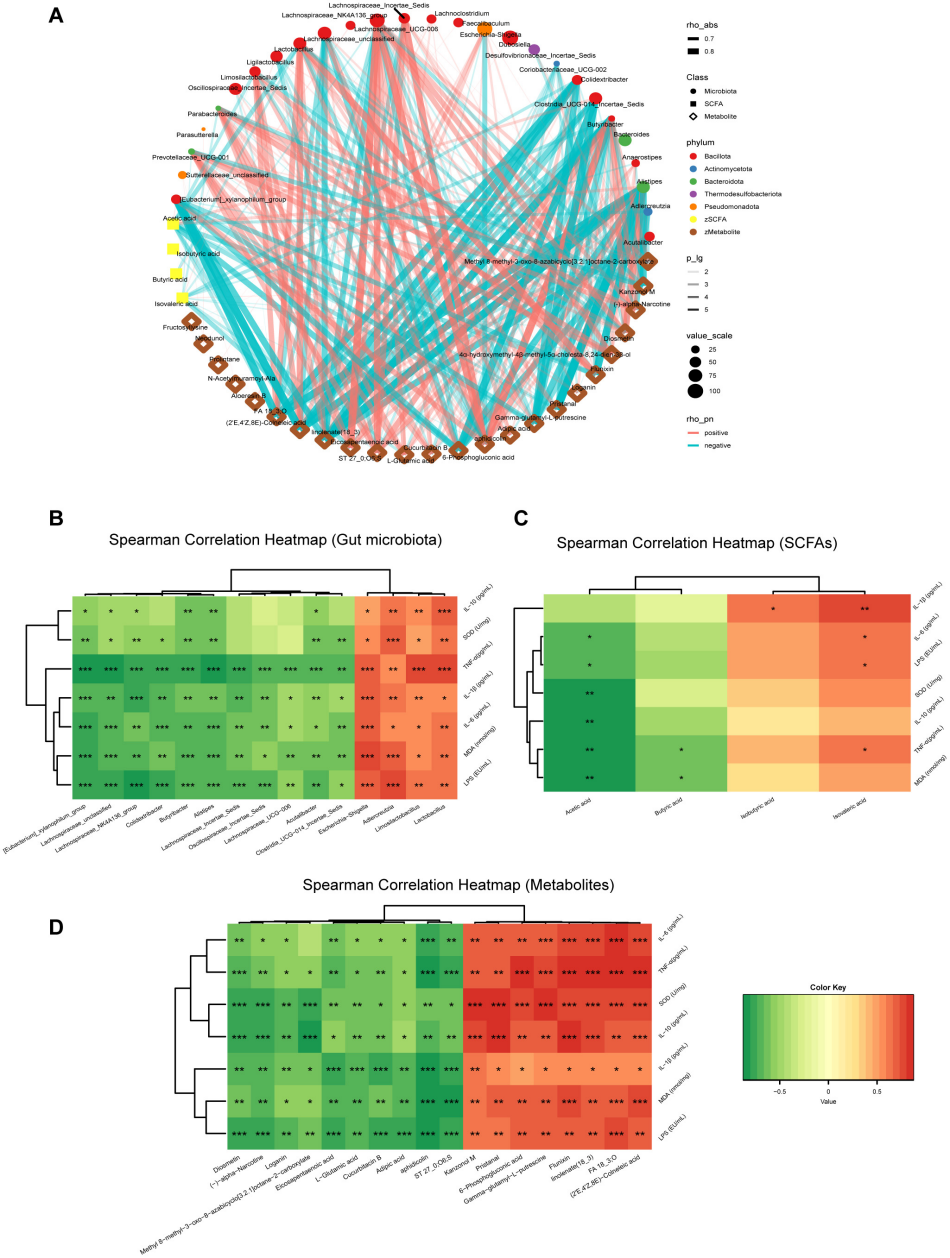
Correlation analysis among gut microbiota, SCFAs, and untargeted metabolomics. **(A)** Spearman correlation network showing significant associations among gut microbial taxa, SCFAs, and metabolites identified by untargeted metabolomics. Node shapes denote microbial taxa (circles), SCFAs (squares), and metabolites (diamonds), with colors indicating microbial phyla or metabolite classes. **Red** and **blue** edges indicate positive and negative correlations, respectively, and edge width reflects the absolute correlation coefficient (|ρ|). Heatmaps illustrating correlations among differential gut microbiota **(B)**, SCFAs **(C)**; as well as differential metabolites from untargeted metabolomics **(D)** with inflammatory factors, LPS, and oxidative stress factors. Color scale indicates correlation direction and strength (**green** to **red**), and asterisks denote statistical significance. *p < 0.05, **p < 0.01, ***p < 0.001.

To further clarify the relationships between gut microbiota and metabolic alterations induced by *F. prausnitzii* intervention, an integrated correlation analysis of differential gut microbiota, SCFAs, fecal metabolites and LPS-associated indices was performed (Figure 9). The correlation network revealed that SCFAs-producing taxa, particularly members of the *Lachnospiraceae* and *Oscillospiraceae* families, were positively correlated with acetate and butyrate, while showing negative associations with branched-chain SCFAs and pro-inflammatory lipid metabolites (Figure 9A). In contrast, inflammation-associated taxa such as *Escherichia–Shigella* exhibited opposite correlation patterns.

Consistent with these findings, correlation heatmaps demonstrated that acetate and butyrate were negatively correlated with LPS exposure and positively associated with *F. prausnitzii* treatment, whereas branched-chain SCFAs showed the opposite trend (Figures 9B, C). Moreover, fecal metabolites involved in inflammatory and oxidative stress-related lipid metabolism were positively correlated with the LPS group but negatively correlated with *F. prausnitzii* intervention (Figure 9D). Collectively, these results demonstrate a tightly interconnected microbiota-metabolite network, indicating that *F. prausnitzii* intervention modulated gut microbial composition and SCFAs production, which were closely linked to changes in fecal metabolic profiles, especially lipid metabolism, inflammation, and oxidative stress related pathways.

## 4 Discussion

Despite rapid advances in elucidating the pathogenesis and developing therapeutic approaches for S-ALI, it remains associated with high morbidity and mortality rates. Bacterial infection is a major causative factor in S-ALI. LPS released from the cell walls of Gram-negative bacteria can induce inflammatory cell infiltration, pro-inflammatory mediator production, and tissue edema and injury (Van Der Poll et al., 2017). In the present study, we identified a potential association between *F. prausnitzii* and sepsis progression in patients with clinical S-ALI. This study further suggest that live *F. prausnitzii* alleviates both CLP- and LPS-induced S-ALI in mice. This protective effect may be related to its ability to modulate gut microbiota composition, enhance short-chain fatty acid production—particularly butyrate—and improve intestinal barrier integrity, thereby reducing circulating LPS levels (Özçam and Lynch, 2024). In addition, our results indicate that *F. prausnitzii* intervention may influence AA-related metabolism and increase levels of its downstream anti-inflammatory mediator LXA4, which may be associated with activation of the Nrf2/HO-1 antioxidant pathway and attenuation of oxidative stress (Luan et al., 2022; Arumugam et al., 2025). Collectively, these findings suggest a potential link between gut microbiota-derived metabolites and host lipid mediator pathways in the regulation of S-ALI

The gut microbiota is widely recognized as a key regulator of local and systemic immunity and is closely associated with pulmonary diseases, particularly through the gut-lung axis, a bidirectional communication network linking intestinal microbiota and lung immune responses (Druszczynska et al., 2024). Gut microbiota plays a crucial role in maintaining the integrity of the immune system, suppressing bacterial translocation in the gut, and alleviating lung injury (Odenwald and Turner, 2017). This conclusion also provides an important theoretical support for the existence of the “gut-lung axis”. In the context of sepsis, however, the gut microbiota homeostasis is impaired, leading to the overgrowth and translocation of pathogenic microbes; these events in turn accelerate the sepsis-associated inflammatory cascade and exacerbate the development of multiple organ dysfunction syndrome (MODS). Our clinical data demonstrated significant gut microbiota dysbiosis in S-ALI patients, particularly involving *F. prausnitzii*. In addition, we observed reduced α-diversity and distinct separation in β-diversity, indicating a restructuring of the microbial community in S-ALI. Our findings are consistent with the anti-inflammatory effects of this bacterium on cytokine production in mouse models of inflammatory bowel disease (Martín et al., 2023). Furthermore, recent studies have indicated that supplementation with *F. prausnitzii* reduces viral load and inflammatory responses during influenza virus infection in mice (Chollet et al., 2024). Our data further indicate that supplementation with *F. prausnitzii* as an intervention can alleviate S-ALI through its anti-inflammatory and immunomodulatory effects, providing an evidence for the role of the gut-lung axis in critical illness.

The intestinal epithelial barrier serves as a critical defense against translocation of harmful substances. In the setting of sepsis, the rate of intestinal epithelial cells (IECs) apoptosis is elevated. Excessive IECs death then induces localized impairment of intestinal permeability and widening of interepithelial gaps, thereby compromising the integrity and function of the gut barrier (Wu et al., 2023; Huang et al., 2025). Studies have demonstrated that in the context of sepsis and multiple organ failure, tight junction protein expression is significantly suppressed, leading to enhanced intestinal epithelial barrier permeability. This alteration facilitates the entry of toxins such as LPS into the body, ultimately amplifying organ inflammation (Xu et al., 2021; Oami et al., 2024). Consistent with previous studies, we observed disruption of intestinal villus architecture, inflammatory cell infiltration, and decreased expression of tight junction proteins (ZO-1, Claudin-4, and Occludin) in S-ALI mice. *F. prausnitzii* treatment restored intestinal structure, reduced inflammation, and significantly increased tight junction protein expression, indicating improved barrier integrity.

The gut microbiota is essential for intestinal physiology, and its dysbiosis is a key contributor to sepsis pathogenesis. Specifically, sepsis drives a shift in the microbial community characterized by depleted symbionts and an expansion of pathobionts, including harmful LPS-producing bacteria. This imbalance not only can trigger sepsis but also perpetuates a vicious cycle that exacerbates disease severity (Jin et al., 2020; Zhou and Liao, 2021). The results of this study indicate that S-ALI mice exhibit that increased abundance of Gram-negative bacteria, particularly *Escherichia–Shigella*, may probably major producers of LPS. These findings are consistent with previous studies demonstrating enrichment of LPS-producing bacteria in sepsis and ALI models (Zhou and Liao, 2021; Li et al., 2025), while our data further highlight specific alterations in key taxa such as *F. prausnitzii*, suggesting a potentially unique regulatory role in S-ALI. Dysbiosis further exacerbates damage to the intestinal mucosal barrier, leading to increased pathogen translocation and heightened susceptibility to infection in distant organs. In sepsis-induced multiple organ dysfunction, LPS derived from gut bacteria plays a key role in both induction and promotion (Li et al., 2024; Qian et al., 2025). Concurrently, increased permeability of the intestinal mucosal barrier leads to elevated levels of LPS in the circulatory system, further exacerbating pulmonary inflammation and injury. *F. prausnitzii* reduced LPS-producing bacteria while promoting beneficial taxa *Lachnospiraceae* and *Akkermansia muciniphila*, thereby mitigating inflammation and restoring microbial balance. *Akkermansia muciniphila* has been demonstrated to produce a novel bioactive tripeptide that effectively suppresses systemic inflammation induced by sepsis (Xie et al., 2024). *Lachnospiraceae bacteria* exerts a promising potential probiotics for treating alcoholic liver disease (ALD) by suppressing oxidative stress, exerting anti-inflammatory effects, and preventing ferroptosis (Zhang H. et al., 2025). In summary, supplementation with *F. prausnitzii* effectively reverses S-ALI-induced gut microbiota imbalance by selectively reshaping microbial composition by inhibiting harmful bacteria while promoting the proliferation of beneficial bacteria, thereby exerting comprehensive positive regulation on both abundance and diversity within the microbial community. These findings highlight that S-ALI-associated dysbiosis occurs at specific taxonomic levels rather than representing a non-specific microbial imbalance.

The partial protective effect of the gut microbiota during S-ALI has been associated with the systemic effects of butyrate, a major kind of SCFAs produced by obligate anaerobic gut bacteria (Wei et al., 2024). SCFAs serve as a nutritional source for intestinal cells. Reduced levels of SCFAs lead to impaired intestinal mucosal barrier function and diminished capacity to resist external pathogens (Peng et al., 2024; Xuan et al., 2024). In addition, butyrate has been shown to enhance antimicrobial activity, improve macrophage function, and modulate inflammatory responses (Schulthess et al., 2019; Zhao et al., 2023). Consistent with previous studies using butyrate pre-treatment, *F. prausnitzii* pre-treatment effectively prevents bacterial pneumonia in mice (Kullberg et al., 2025). *F. prausnitzii* and its metabolite butyrate suppress ferroptosis by downregulating LCN2 expression in aged cardiomyocytes, thereby alleviating age-related heart failure (Zhang Y. et al., 2025). In the present study, *F. prausnitzii* supplementation was associated with increased fecal butyrate levels, which may contribute to its protective effects. However, the specific role of butyrate was not directly investigated in this study and therefore should be interpreted with caution. Furthermore, our findings further suggest that the protective effects of *F. prausnitzii* are not solely dependent on butyrate but may involve additional metabolic pathways, such as AA metabolism.

When stimulated or damaged, AA is released from the cell membrane through the action of phospholipase A2 (Glaser et al., 2025). Once released, AA generates a large number of metabolites through the action of cyclooxygenase, lipoxygenase, and cytochrome P450 (Hu et al., 2024). AA metabolites are generally considered as inflammatory bioactive lipids or eicosanoids. However, certain lipoxygenase products (such as lipoxins) are potent anti-inflammatory mediators (Suchitha et al., 2024). Notably, *F. prausnitzii* intervention increased levels of LXA4, a specialized pro-resolving lipid mediator derived from AA (Chiang and Serhan, 2020). LXA4 is known to suppress excessive inflammation and promote resolution of tissue injury, particularly in the lung (Sousa et al., 2025). The observed increase in LXA4 provides a mechanistic bridge between gut microbial modulation and attenuation of pulmonary inflammation.

Oxidative stress is a hallmark of S-ALI and a major contributor to alveolar damage. *F. prausnitzii* enhanced antioxidant capacity, as evidenced by increased SOD activity and reduced MDA levels, which may be associated with the activation of the Nrf2-HO-1 signaling pathway. Nrf2 is a master regulator of cellular redox homeostasis, and its downstream target HO-1 plays a critical role in protecting against oxidative and inflammatory injury (Huang et al., 2024). The coordinated upregulation of LXA4 and Nrf2/HO-1 signaling suggests that *F. prausnitzii* may promote a pro-resolving, antioxidant milieu that limits lung damage during sepsis. Together, these findings indicate a coordinated anti-inflammatory and antioxidative mechanistic effect potentially associated with AA metabolism and the Nrf2-HO-1 signaling pathway. In addition, classical NF-κB/MAPK/JNK inflammatory signaling pathways are known to play important roles in the pathogenesis of acute lung injury and inflammatory response. These pathways are closely interconnected with oxidative stress signaling and play key roles in mediating inflammation (Sies and Jones, 2020; Zhang et al., 2021; Liu et al., 2025). However, their specific roles involved in the protective effects of *F. prausnitzii* are not directly examined in the present study and needed to warrant further investigation.

This study has several limitations. The relatively small clinical cohort limits generalizability, despite observed associations between gut microbiota alterations and clinical indicators. In addition, mechanistic insights derived from CLP- and LPS-induced models may not fully reflect the complexity of human S-ALI. A further limitation of this study is that the specific role of butyrate in mediating the protective effects of *F. prausnitzii* was not directly validated. Moreover, although our findings suggest the involvement of AA metabolism and Nrf2-HO-1 signaling pathway, the causal role of the AA-LXA4-Nrf2-HO-1 axis remains to be further validated using pharmacological inhibition. Further investigation should be performed to define causal links within the gut microbiota-metabolite-host axis. Clinical translational potential of *F. prausnitzii* particularly remains meeting challenges for rigorous evaluation of safety, dosing, and efficacy.

Collectively, our findings support a mechanistic model in which *F. prausnitzii* protects against S-ALI by coordinating the gut-lung axis and linking microbial metabolism to host lipid mediator signaling and antioxidant defense. These results highlight the therapeutic potential of targeting specific commensal bacteria to mitigate sepsis-induced organ injury.

## 5 Conclusion

**Figure 10:**
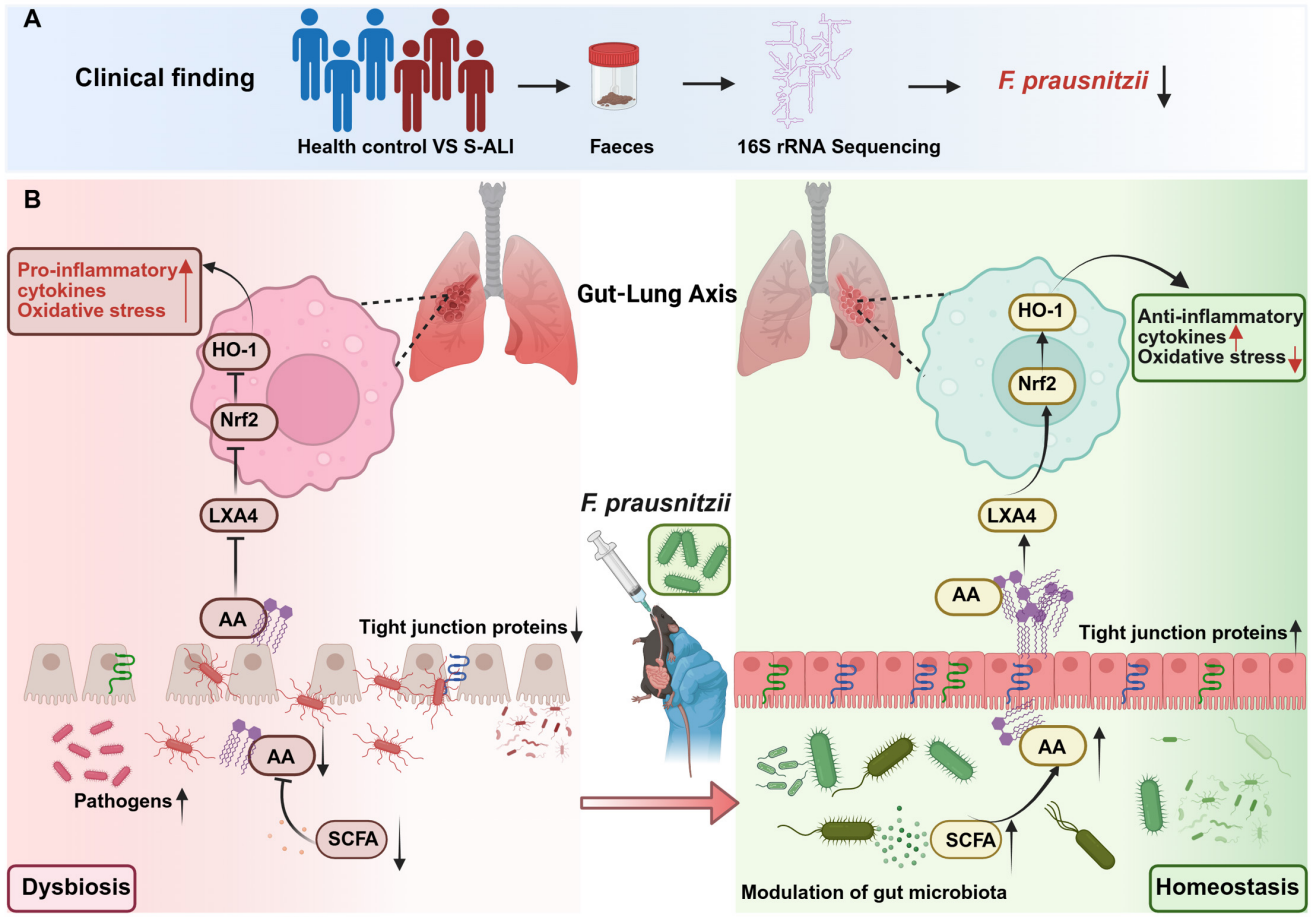
Mechanism of *F. prausnitzii* in alleviating sepsis-induced lung injury via the gut-lung axis. **(A)** Clinical 16S rRNA sequencing reveals depletion of *F. prausnitzii* in patients with S-ALI. **(B)** Pre-treatment with *F. prausnitzii* via the gut-lung axis regulates the AA-LXA4-Nrf2-HO-1 pathway and restores gut microecology, ultimately reducing pulmonary inflammation, oxidative stress, and vascular permeability, and alleviating S-ALI.

This study demonstrates that *F. prausnitzii* plays a critical protective role in S-ALI through regulation of the gut-lung axis. Patients with S-ALI exhibited marked gut microbiota dysbiosis characterized by a significant reduction in *F. prausnitzii*, which was positively correlated with systemic inflammation and disease severity (Figure 10A). In both CLP- and LPS-induced murine models of S-ALI, *F. prausnitzii* supplementation effectively attenuated S-ALI, accompanied by a pronounced reduction in inflammatory responses and oxidative stress. These protective effects were closely associated with restoration of gut microecological homeostasis, including reshaping of gut microbial composition, enhancement of intestinal barrier integrity, and increased production of microbial-derived short-chain fatty acids, particularly butyrate. Mechanistically, integrated untargeted metabolomics and correlation analyses revealed that *F. prausnitzii* intervention modulated AA metabolism and may be associated with the LXA4-Nrf2-HO-1 signaling pathway, which is associated with inflammation and oxidative stress (Figure 10B). Collectively, these findings highlight *F. prausnitzii* and its metabolite butyrate as promising microbiota-targeted therapeutic candidates for the prevention and treatment of S-ALI via modulation of the gut-lung axis and lipid metabolic pathways.

## Data Availability

The raw sequencing data have been uploaded to the NCBI Sequence Read Archive (SRA) with the accession number PRJNA1417153.
