# Supplementary material for: Faecalibacterium prausnitzii ameliorates sepsis-induced acute lung injury via gut-lung axis by regulating arachidonic acid-LXA4-Nrf2-HO-1 pathway and restoring gut microecology
Source: Front Microbiol. 2026 May 28;17:1820338. doi: 10.3389/fmicb.2026.1820338 (PMC13255568; doi:10.3389/fmicb.2026.1820338)
Supplement: Supplementary file 1 [file Data_Sheet_1.docx]

**Manuscript ID:** 1820338

**Title:** *Faecalibacterium prausnitzii* ameliorates sepsis-induced acute lung injury via gut-lung axis by regulating arachidonic acid-LXA4-Nrf2-HO-1 pathway and restoring gut microecology.

**Journal:** Frontiers in Microbiology

Supplementary Material

**1 Supplementary Table**

**1.1 Supplementary Table S1. Comparison of the general information between S-ALI Group and HC Group.**

| **Indicator** | **S-ALI (n = 15)** | **HC (n = 10)** | **p-value** |
| --- | --- | --- | --- |
| Age, yr [median (P25, P75)] | 65.00 (54.00, 72.00) | 57.00 (54.00, 60.00) | 0.071 |
| Sex, n (%) |  |  | 0.378 |
| Male | 12 (80.0%) | 6 (60.0%) |  |
| Female | 3 (20.0%) | 4 (40.0%) |  |
| BMI, kg/m^2 [median (P25, P75)] | 23.91 (20.55, 26.77) | 30.15 (27.88, 31.21) | <0.001 |
| Underlying disease, cases |  |  | 1.000 |
| No | 8 (53.3%) | 5 (50.0%) |  |
| Yes | 7 (46.7%) | 5 (50.0%) |  |
| ALB, g/L [median (P25, P75)] | 26.50 (24.10, 29.40) | - | - |
| APACHE II score [median (P25, P75)] | 21.00 (17.00, 24.00) | - | - |
| SOFA score [median (P25, P75)] | 8.00 (5.00, 9.00) | - | - |

Data are presented as median (P25, P75) or n (%).

For variables with no available data in one group, the statistic and p value were not calculated and are shown as '-'.

Continuous variables were compared using nonparametric tests. Categorical variables were analyzed using the chi-square test or Fisher’s exact test.

**1.2 Supplementary Table S2. Relative abundance (mean ± SEM) of key taxa across taxonomic levels in clinical samples.**

| **Taxa** | **S-ALI**  **(mean ± SEM)** | **HC**  **(mean ± SEM)** | **p-value** |
| --- | --- | --- | --- |
| **Phylum** | | | |
| *Actinobacteriota* | 0.0125±0.0048 | 0.0301±0.0088 | 0.0283 |
| *Cyanobacteria* | 0±0 | 0.0004±0.0003 | 0.0307 |
| *Patescibacteria* | 0.0001±0.0001 | 0.0002±0.0001 | 0.0349 |
| **Class** | | | |
| *Clostridia* | 0.1952±0.0526 | 0.4744±0.0593 | 0.0044 |
| *Negativicutes* | 0.0050±0.0021 | 0.0693±0.0110 | <0.0001 |
| *Actinobacteria* | 0.0091±0.0038 | 0.0267±0.0082 | 0.0315 |
| **Order** | | | |
| *Oscillospirales* | 0.1006±0.0391 | 0.2141±0.0434 | 0.0158 |
| *Lachnospirales* | 0.0777±0.0378 | 0.2203±0.0468 | 0.0025 |
| *Pseudomonadales* | 0.0701±0.0651 | 0±0 | 0.0082 |
| *Veillonellales-Selenomonadales* | 0.0042±0.0020 | 0.0424±0.0145 | 0.0083 |
| *Bifidobacteriales* | 0.0063±0.0030 | 0.0266±0.0082 | 0.0094 |
| *Acidaminococcales* | 0.0008±0.0004 | 0.0268±0.0065 | 0.0019 |
| *Burkholderiales* | 0.0068±0.0049 | 0.0111±0.0044 | 0.0212 |
| *Clostridiales* | 0.0020±0.0019 | 0.0021±0.0006 | 0.0034 |
| *Monoglobales* | 0.0001±0.0001 | 0.0014±0.0006 | 0.0031 |
| **Genus** | | | |
| *Enterococcus* | 0.1651±0.0851 | 0.0001±0.0001 | 0.0043 |
| *Acinetobacter* | 0.0700±0.0651 | 0±0 | 0.0157 |
| *Faecalibacterium* | 0.0126±0.0091 | 0.0592±0.0128 | 0.0032 |
| *Lachnospiraceae_unclassified* | 0.0316±0.0271 | 0.0238±0.0053 | 0.0065 |
| *Subdoligranulum* | 0.0042±0.0014 | 0.0399±0.0101 | 0.0013 |
| *Roseburia* | 0.0006±0.0003 | 0.0369±0.0112 | 0.0002 |
| *Bifidobacterium* | 0.0063±0.0030 | 0.0266±0.0082 | 0.0094 |
| *Ruminococcaceae_CAG-352* | 0.0026±0.0024 | 0.0300±0.0168 | 0.0359 |
| *Lachnospira* | 0.0002±0.0001 | 0.0289±0.0132 | <0.0001 |
| *Phascolarctobacterium* | 0.0008±0.0004 | 0.0268±0.0065 | 0.0019 |

| **Taxa** | **CON (mean ± SEM)** | **LPS (mean ± SEM)** | **LPS_FP (mean ± SEM)** | **FP(mean ± SEM)** | **p-value** |
| --- | --- | --- | --- | --- | --- |
| **Phylum** | | | | | |
| *Bacillota* | 0.5793±0.0345 | 0.3021±0.0371 | 0.5913±0.0818 | 0.4791±0.0553 | 0.0093 |
| *Pseudomonadota* | 0.0063±0.0012 | 0.3021±0.0976 | 0.0079±0.0068 | 0.0002±0.0001 | 0.0007 |
| *Thermodesulfobacteriota* | 0.0047±0.0019 | 0.0197±0.0042 | 0.0214±0.0048 | 0.0149±0.0088 | 0.0267 |
| *Actinomycetota* | 0.0088±0.0039 | 0.0213±0.0051 | 0.0188±0.0041 | 0.0038±0.0011 | 0.0417 |
| *Cyanobacteriota* | 0.0012±0.0002 | 0.0001±0.0001 | 0.0001±0.0001 | 0.0015±0.0004 | 0.0011 |
| *Deferribacterota* | 0.0004±0.0001 | 0±0 | 0±0 | 0±0 | 0.0011 |
| **Genus** | | | | | |
| *Dubosiella* | 0.1134±0.0541 | 0.0151±0.0048 | 0.3829±0.0825 | 0.0914±0.0336 | 0.0030 |
| *Escherichia-Shigella* | 0.0004±0.0001 | 0.2895±0.1004 | 0.0079±0.0068 | <0.0001 | 0.0007 |
| *Lachnospiraceae_unclassified* | 0.0422±0.0068 | 0.0165±0.0068 | 0.0202±0.0044 | 0.0670±0.0124 | 0.0075 |
| *Roseburia* | 0.0047±0.0011 | 0.0005±0.0003 | 0.0013±0.0005 | 0.0035±0.0011 | 0.0079 |
| *Akkermansia* | 0.0690±0.0213 | 0.0415±0.0358 | 0.1148±0.0438 | 0.0560±0.0132 | 0.1529 |

**1.3 Supplementary Table S3. Relative abundance (mean ± SEM) of key taxa across taxonomic levels in murine samples.**

**2 Supplementary Figure**

**2.1 Supplementary FigureS1**

**
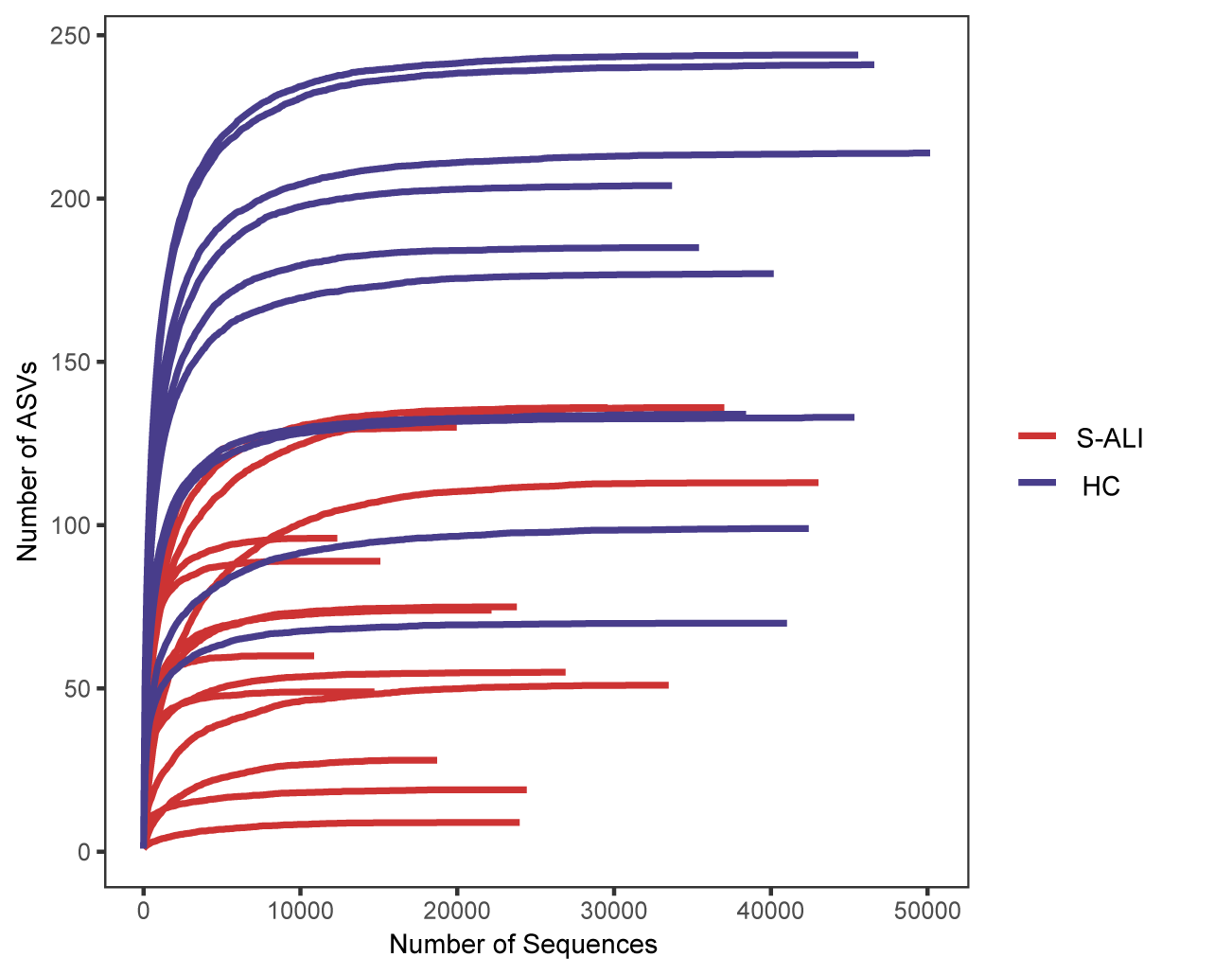
**

**Figure S1.** Rarefaction curves of 16S rRNA sequencing for clinical fecal samples. Rarefaction curves of clinical fecal samples showing ASV counts at varying sequencing depths. The curves indicate sufficient sequencing depth to capture the majority of microbial diversity in both groups.

**2.2 Supplementary Figure S2**

**
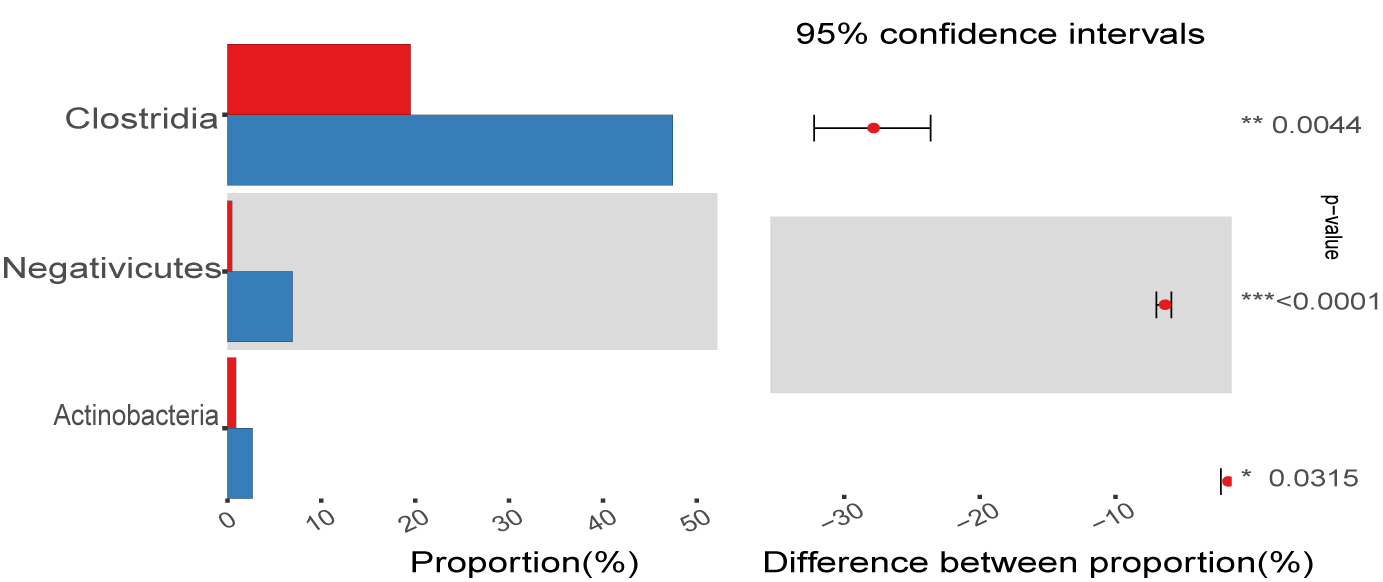
**

**Figure S2.** Class-level taxonomic abundance in clinical fecal samples. Relative abundance and proportion differences at the class level with 95% confidence intervals. Statistically significant differences are indicated.*p < 0.0001, p = 0.0044, p = 0.0315.

**2.3 Supplementary Figure S3:**

**
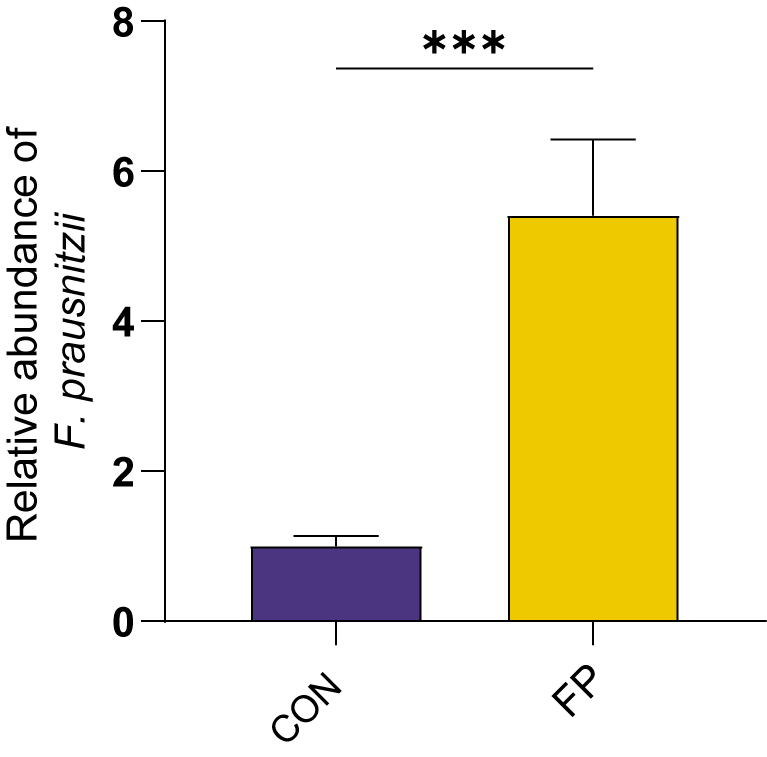
**

**Figure S3.** Confirmation of *F. prausnitzii* colonization in the gut. Colonization of *F. prausnitzii* was verified by qPCR analysis of fecal samples (n = 3–4 per group). The relative abundance of F. prausnitzii was quantified using species-specific primers and normalized to total bacterial 16S rRNA levels. *** p < 0.001.

**2.4 Supplementary Figure S4**

**
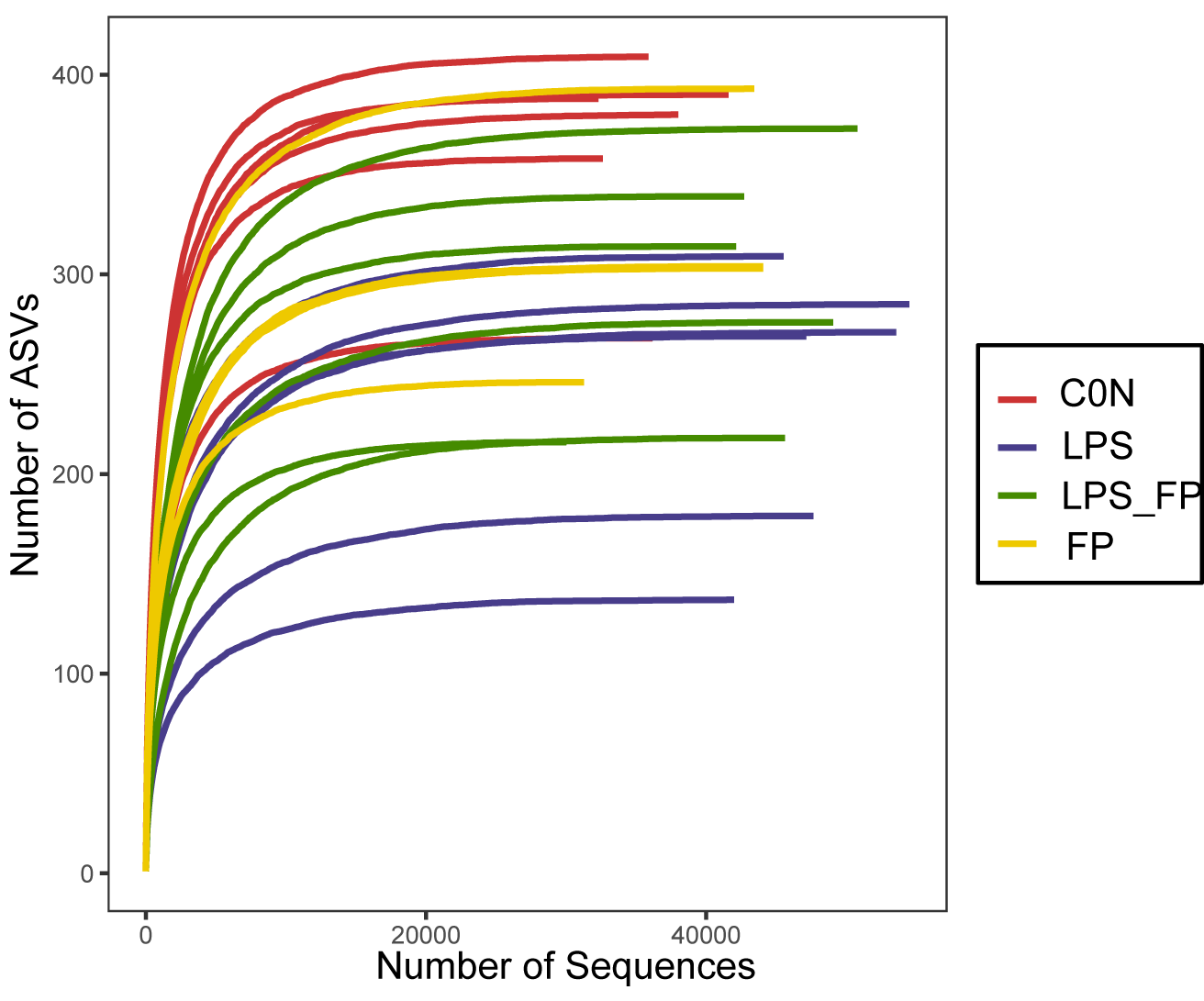
**

**Figure S4.** Mouse fecal sample rarefaction curves. Rarefaction curves showing the number of ASVs as a function of sequencing depth for mouse groups. The X-axis represents sequence numbers, and the Y-axis represents ASVs detected. The curves indicate sufficient sequencing depth to capture microbial diversity across all groups.
